# A genome-wide association study of marginal zone lymphoma shows association to the HLA region

**DOI:** 10.1038/ncomms6751

**Published:** 2015-01-08

**Authors:** Joseph Vijai, Zhaoming Wang, Sonja I. Berndt, Christine F. Skibola, Susan L. Slager, Silvia de Sanjose, Mads Melbye, Bengt Glimelius, Paige M. Bracci, Lucia Conde, Brenda M. Birmann, Sophia S. Wang, Angela R. Brooks-Wilson, Qing Lan, Paul I. W. de Bakker, Roel C. H. Vermeulen, Carol Portlock, Stephen M. Ansell, Brian K. Link, Jacques Riby, Kari E. North, Jian Gu, Henrik Hjalgrim, Wendy Cozen, Nikolaus Becker, Lauren R. Teras, John J. Spinelli, Jenny Turner, Yawei Zhang, Mark P. Purdue, Graham G. Giles, Rachel S. Kelly, Anne Zeleniuch-Jacquotte, Maria Grazia Ennas, Alain Monnereau, Kimberly A. Bertrand, Demetrius Albanes, Tracy Lightfoot, Meredith Yeager, Charles C. Chung, Laurie Burdett, Amy Hutchinson, Charles Lawrence, Rebecca Montalvan, Liming Liang, Jinyan Huang, Baoshan Ma, Danylo J. Villano, Ann Maria, Marina Corines, Tinu Thomas, Anne J. Novak, Ahmet Dogan, Mark Liebow, Carrie A. Thompson, Thomas E. Witzig, Thomas M. Habermann, George J. Weiner, Martyn T. Smith, Elizabeth A. Holly, Rebecca D. Jackson, Lesley F. Tinker, Yuanqing Ye, Hans-Olov Adami, Karin E. Smedby, Anneclaire J. De Roos, Patricia Hartge, Lindsay M. Morton, Richard K. Severson, Yolanda Benavente, Paolo Boffetta, Paul Brennan, Lenka Foretova, Marc Maynadie, James McKay, Anthony Staines, W. Ryan Diver, Claire M. Vajdic, Bruce K. Armstrong, Anne Kricker, Tongzhang Zheng, Theodore R. Holford, Gianluca Severi, Paolo Vineis, Giovanni M. Ferri, Rosalia Ricco, Lucia Miligi, Jacqueline Clavel, Edward Giovannucci, Peter Kraft, Jarmo Virtamo, Alex Smith, Eleanor Kane, Eve Roman, Brian C. H. Chiu, Joseph F. Fraumeni, Xifeng Wu, James R. Cerhan, Kenneth Offit, Stephen J. Chanock, Nathaniel Rothman, Alexandra Nieters

**Affiliations:** 1 Department of Medicine, Memorial Sloan-Kettering Cancer Center, New York, New York 10065, USA; 2 Cancer Genomics Research Laboratory, Division of Cancer Epidemiology and Genetics, National Cancer Institute, Gaithersburg, Maryland 20877, USA; 3 Division of Cancer Epidemiology and Genetics, National Cancer Institute, Bethesda, Maryland 20892, USA; 4 Department of Epidemiology, School of Public Health and Comprehensive Cancer Center, Birmingham, Alabama 35233, USA; 5 Division of Environmental Health Sciences, University of California Berkeley School of Public Health, Berkeley, California 94720, USA; 6 Department of Health Sciences Research, Mayo Clinic, Rochester, Minnesota 55905, USA; 7 Unit of Infections and Cancer (UNIC), Cancer Epidemiology Research Programme, Institut Catala d’Oncologia, IDIBELL, Barcelona 8907, Spain; 8 Centro de Investigación Biomédica en Red de Epidemiología y Salud Pública (CIBERESP), Barcelona 8036, Spain; 9 Department of Epidemiology Research, Division of Health Surveillance and Research, Statens Serum Institut, Copenhagen 2300, Denmark; 10 Department of Medicine, Stanford University School of Medicine, Stanford, California 94305, USA; 11 Department of Oncology and Pathology, Karolinska Institutet, Karolinska University Hospital Solna, Stockholm 17176, Sweden; 12 Department of Radiology, Oncology and Radiation Science, Uppsala University, Uppsala 75105, Sweden; 13 Department of Epidemiology & Biostatistics, University of California San Francisco, San Francisco, California 94118, USA; 14 Channing Division of Network Medicine, Department of Medicine, Brigham and Women’s Hospital and Harvard Medical School, Boston, Massachusetts 02115, USA; 15 Department of Cancer Etiology, City of Hope Beckman Research Institute, Duarte, California 91030, USA; 16 Genome Sciences Centre, BC Cancer Agency, Vancouver, British Columbia, Canada V5Z1L3; 17 Department of Biomedical Physiology and Kinesiology, Simon Fraser University, Burnaby, British Columbia, Canada V5A1S6; 18 Department of Medical Genetics, Center for Molecular Medicine, University Medical Center Utrecht, Utrecht 3584 CG, The Netherlands; 19 Department of Epidemiology, Julius Center for Health Sciences and Primary Care, University Medical Center Utrecht, Utrecht 3584 CX, The Netherlands; 20 Institute for Risk Assessment Sciences, Utrecht University, Utrecht 3508 TD, The Netherlands; 21 Department of Medicine, Mayo Clinic, Rochester, Minnesota 55905, USA; 22 Department of Internal Medicine, Carver College of Medicine, The University of Iowa, Iowa City, Iowa 52242, USA; 23 Department of Epidemiology, University of North Carolina at Chapel Hill, Chapel Hill, North Carolina 27599, USA; 24 Carolina Center for Genome Sciences, University of North Carolina at Chapel Hill, Chapel Hill, North Carolina 27599, USA; 25 Department of Epidemiology, M.D. Anderson Cancer Center, Houston, Texas 77030, USA; 26 Department of Preventive Medicine, USC Keck School of Medicine, University of Southern California, Los Angeles, California 90033, USA; 27 Norris Comprehensive Cancer Center, USC Keck School of Medicine, University of Southern California, Los Angeles, California 90033, USA; 28 Division of Cancer Epidemiology, German Cancer Research Center (DKFZ), Heidelberg 69120, Germany; 29 Epidemiology Research Program, American Cancer Society, Atlanta, Georgia 30303, USA; 30 Cancer Control Research, BC Cancer Agency, Vancouver, British Columbia, Canada V5Z1L3; 31 School of Population and Public Health, University of British Columbia, Vancouver, British Columbia, Canada V6T1Z3; 32 Pathology, Australian School of Advanced Medicine, Macquarie University, Sydney, New South Wales 2109, Australia; 33 Department of Histopathology, Douglass Hanly Moir Pathology, Macquarie Park, New South Wales 2113, Australia; 34 Department of Environmental Health Sciences, Yale School of Public Health, New Haven, Connecticut 06520, USA; 35 Cancer Epidemiology Centre, Cancer Council Victoria, Melbourne, Victoria 3053, Australia; 36 Centre for Epidemiology and Biostatistics, Melbourne School of Population and Global Health, University of Melbourne, Carlton, Victoria 3010, Australia; 37 MRC-PHE Centre for Environment and Health, School of Public Health, Imperial College London, London W2 1PG, UK; 38 Department of Population Health, New York University School of Medicine, New York, New York 10016, USA; 39 Cancer Institute, New York University School of Medicine, New York, New York 10016, USA; 40 Department of Biomedical Science, University of Cagliari, Monserrato, Cagliari 09042, Italy; 41 Environmental Epidemiology of Cancer Group, Inserm, Centre for research in Epidemiology and Population Health (CESP), U1018, Villejuif F-94807, France; 42 UMRS 1018, Univ Paris Sud, Villejuif F-94807, France; 43 Registre des hémopathies malignes de la Gironde, Institut Bergonié, Bordeaux 33076, France; 44 Department of Epidemiology, Harvard School of Public Health, Boston, Massachusetts 02115, USA; 45 Department of Health Sciences, University of York, York YO10 5DD, UK; 46 Health Studies Sector, Westat, Rockville, Maryland 20850, USA; 47 Department of Biostatistics, Harvard School of Public Health, Boston, Massachusetts 02115, USA; 48 College of Information Science and Technology, Dalian Maritime University, Dalian 116026, China; 49 Departments of Laboratory Medicine and Pathology, Memorial Sloan-Kettering Cancer Center, New York, New York 10065, USA; 50 Division of Endocrinology, Diabetes and Metabolism, The Ohio State University, Columbus, Ohio 43210, USA; 51 Division of Public Health Sciences, Fred Hutchinson Cancer Research Center, Seattle, Washington 98117, USA; 52 Department of Medical Epidemiology and Biostatistics, Karolinska Institutet, Stockholm 17177, Sweden; 53 Department of Medicine Solna, Karolinska Institutet, Stockholm 17176, Sweden; 54 Department of Environmental and Occupational Health, Drexel University School of Public Health, Philadelphia, Pennsylvania 19104, USA; 55 Department of Family Medicine and Public Health Sciences, Wayne State University, Detroit, Michigan 48201, USA; 56 The Tisch Cancer Institute, Icahn School of Medicine at Mount Sinai, New York, New York 10029, USA; 57 Group of Genetic Epidemiology, Section of Genetics, International Agency for Research on Cancer, Lyon 69372, France; 58 Department of Cancer Epidemiology and Genetics, Masaryk Memorial Cancer Institute and MF MU, Brno 65653, Czech Republic; 59 EA 4184, Registre des Hémopathies Malignes de Côte d’Or, University of Burgundy and Dijon University Hospital, Dijon 21070, France; 60 Genetic Cancer Susceptibility Group, Section of Genetics, International Agency for Research on Cancer, Lyon 69372, France; 61 School of Nursing and Human Sciences, Dublin City University, Dublin 9, Ireland; 62 Prince of Wales Clinical School, University of New South Wales, Sydney, New South Wales 2052, Australia; 63 Sydney School of Public Health, The University of Sydney, Sydney, New South Wales 2006, Australia; 64 Department of Biostatistics, Yale School of Public Health, New Haven, Connecticut 06520, USA; 65 Human Genetics Foundation, Turin 10126, Italy; 66 Interdisciplinary Department of Medicine, University of Bari, Bari 70124, Italy; 67 Department of Pathological Anatomy, University of Bari, Bari 70124, Italy; 68 Environmental and Occupational Epidemiology Unit, Cancer Prevention and Research Institute (ISPO), Florence 50139, Italy; 69 Department of Nutrition, Harvard School of Public Health, Boston, Massachusetts 02115, USA; 70 Department of Chronic Disease Prevention, National Institute for Health and Welfare, Helsinki FI-00271, Finland; 71 Department of Health Studies, University of Chicago, Chicago, Illinois 60637, USA; 72 Center For Chronic Immunodeficiency, University Medical Center Freiburg, Freiburg 79108, Germany

## Abstract

Marginal zone lymphoma (MZL) is the third most common subtype of B-cell non-Hodgkin lymphoma. Here we perform a two-stage GWAS of 1,281 MZL cases and 7,127 controls of European ancestry and identify two independent loci near *BTNL2* (rs9461741, *P*=3.95 × 10^−15^) and *HLA-B* (rs2922994, *P*=2.43 × 10^−9^) in the HLA region significantly associated with MZL risk. This is the first evidence that genetic variation in the major histocompatibility complex influences MZL susceptibility.

## Abstract (web summary)

Marginal zone lymphoma (MZL) is a common subtype of B-cell non-Hodgkin lymphoma. Here the authors carry out a two-stage genome-wide association study in over 8,000 Europeans and identify two new MZL risk loci at chromosome 6p, implicating the major histocompatibility complex in the disease for the first time.

## Graphical abstract

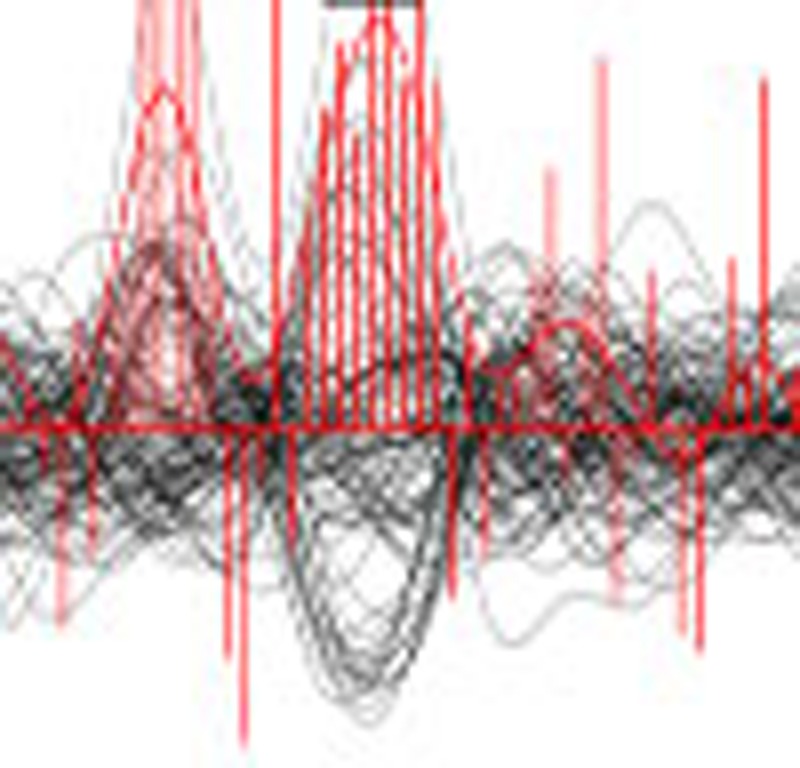

Marginal zone lymphoma (MZL) encompasses a group of lymphomas that originate from marginal zone B cells present in extranodal tissue and lymph nodes. Three subtypes of MZL have been defined, extranodal MZL of mucosa-associated lymphoid tissue (MALT), splenic MZL and nodal MZL, which together account for 7–12% of all non-Hodgkin lymphoma (NHL) cases. Geographic differences in incidence have been observed^1^, and inflammation, immune system dysregulation and infectious agents, such as *Helicobacter pylori*, have been implicated particularly for the gastric MALT subtype^2^, but little else is known of MZL aetiology.

Here we perform the first two-stage, subtype-specific genome-wide association study (GWAS) of MZL and identify two independent single-nucleotide polymorphisms (SNPs) within the HLA region associated with MZL risk. Together with recent studies on other common subtypes of NHL, these results point to shared susceptibility loci for lymphoma in the HLA region.

## Results

### Stage 1 MZL GWAS

As part of a larger NHL GWAS, 890 MZL cases and 2,854 controls from 22 studies in the United States and Europe (Supplementary Table 1) were genotyped using the Illumina OmniExpress array. Genotype data from the Illumina Omni2.5 was also available for 3,536 controls from three of the 22 studies^3^. After applying rigorous quality control filters (Supplementary Table 2, Methods), data for 611,856 SNPs with minor allele frequency (MAF)>1% in 825 cases and 6,221 controls of European ancestry (Supplementary Fig. 1) remained for the stage 1 analysis (Supplementary Table 3). To discover variants associated with risk, logistic regression analysis was performed on these SNPs adjusting for age, gender and three significant eigenvectors computed using principal components analysis (Supplementary Fig. 2, Methods). Examination of the quantile–quantile (Q–Q) plot (Supplementary Fig. 3) showed minimal detectable evidence for population substructure (*λ*=1.01) with some excess of small *P* values. A Manhattan plot revealed association signals at the HLA region (Supplementary Fig. 4; 6p21.33:31,061,211–32,620,572) on chromosome 6 reaching genome-wide significance. Removal of all SNPs in the HLA region resulted in an attenuation of the excess of small *P* values observed in the Q–Q plot, although some excess still remained. To further explore associations within the HLA region and identify other regions potentially associated with risk, common SNPs available in the 1000 Genomes project data release 3 were imputed (Methods).

### Stage 2 genotyping

**Figure 1:**
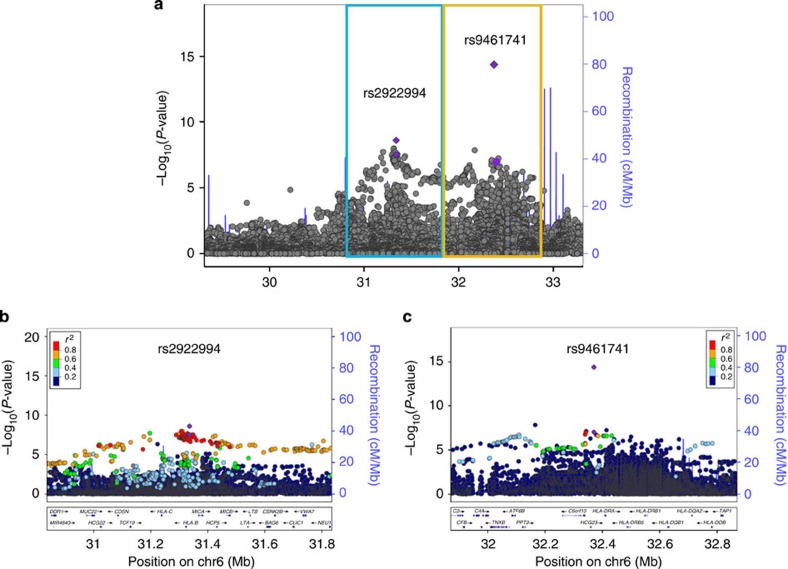
Regional plot showing the HLA associations with MZL. The figure shows the association log_10_ *P* values from the log-additive genetic model for all SNPs in the region from stage 1 (dots) (*n*=825 cases, *n*=6,221 controls) and the log_10_ *P* values from the log-additive genetic model for both stage 1 and 2 combined (purple diamonds) for rs2922994 (*n*=1,230 cases, *n*=7,053 controls) and rs9461741 (*n*=1,277 cases, *n*=7,097 controls). The purple dots show the log_10_ *P* values of these SNPs in stage 1. Top panel (**a**) shows the region encompassing both SNPs. Bottom panel (**b**) regional plot of the most significant SNP rs2922994 at 6p21.33 (**c**) and rs9461741 at 6p21.32. The colours of the dots reflect the LD (as measured by *r*^2^) with the most significant SNP as shown in the legend box.

Ten SNPs in promising loci with *P*≤7.5 × 10^−6^ in the stage 1 discovery were selected for replication (stage 2) in an additional 456 cases and 906 controls of European ancestry (Supplementary Tables 1 and 3). Of the SNPs selected for replication, two SNPs were directly genotyped on the OmniExpress, while the remaining eight were imputed with high accuracy (median info score=0.99) in stage 1 (Supplementary Table 4). Replication was carried out using Taqman genotyping. In the combined meta-analysis of 1,281 cases and 7,127 controls, we identified two distinct loci (Table 1, Fig. 1, Supplementary Table 4) at chromosomes 6p21.32 and 6p21.33 that reached the threshold of genome-wide statistical significance (*P*<5 × 10^−8^). These are rs9461741 in the butyrophilin-like 2 (MHC class II associated) (*BTNL2*) gene at 6p21.32 in HLA class II (*P*=3.95 × 10^−15^, odds ratio (OR)=2.66, confidence interval (CI)=2.08–3.39) and rs2922994 at 6p21.33 in HLA class I (*P*=2.43 × 10^−9^, OR=1.64, CI=1.39–1.92). These two SNPs were weakly correlated (*r*^2^=0.008 in 1000 Genomes CEU population), and when both were included in the same statistical model, both SNPs remained strongly associated with MZL risk (rs9461741, *P*=2.09 × 10^−15^; rs2922994, *P*=6.03 × 10^−10^), suggesting that the two SNPs are independent. Both SNPs were weakly correlated with other SNPs in the HLA region previously reported to be associated with other NHL subtypes or Hodgkin lymphoma (*r*^2^<0.14 for all pairwise comparisons). None of the previously reported SNPs were significantly associated with MZL risk after adjustment for multiple testing (*P*<0.0025) in our study, suggesting the associations are subtype-specific (Supplementary Table 5). Another SNP rs7750641 (*P*=3.34 × 10^−8^; Supplementary Table 4) in strong linkage disequilibrium (LD) with rs2922994 (*r*^2^=0.85) also showed promising association with MZL risk. rs7750641 is a missense variant in transcription factor 19 (*TCF19*), which encodes a DNA-binding protein implicated in the transcription of genes during the G1–S transition in the cell cycle^4^. The non-HLA SNPs genotyped in stage 2 were not associated with MZL risk (Supplementary Table 4).

### HLA alleles

To obtain additional insight into plausible functional variants, we imputed the classical HLA alleles and amino acid residues using SNP2HLA^5^ (Methods). No imputed HLA alleles or amino acid positions reached genome-wide significance (Supplementary Table 6). However, for HLA class I, the most promising associations were observed with HLA-B*08 (*P*=7.94 × 10^−8^), HLA-B*08:01 (*P*=7.79 × 10^−8^) and the *HLA-B* allele encoding an aspartic acid residue at position 9 (Asp9) (*P*=7.94 × 10^−8^), located in the peptide binding groove of the protein. HLA-B*08:01 and Asp9 are highly correlated (*r*^2^≥0.99), and thus their effect sizes were identical (OR=1.67, 95% CI: 1.38–2.01). They are both also in strong LD with rs2922994 (*r*^2^=0.97). Due to the fact that they are collinear, the effects of the SNPs and alleles were indistinguishable from one another in conditional modelling. For HLA class II, a suggestive association was observed with HLA-DRB1*01:02 (OR=2.24, 95% CI: 1.64–3.07, *P*=5.08 × 10^−7^; Supplementary Table 6), which is moderately correlated with rs9461741 (*r*^2^=0.69). Conditional analysis revealed that the effects of rs9461741 (the intragenic SNP in *BTNL2*) and HLA-DRB1*01:02 were indistinguishable statistically (stage 1: rs9461741, *P*_adjusted_=0.064 and HLA-DRB1*01:02, *P*_adjusted_=0.29). A model containing both HLA-B*08:01 and HLA-DRB1*01:02 showed that the two alleles were independent (HLA-B*08:01: *P*_adjusted_=4.65 × 10^−8^ and HLA-DRB1*01:02: *P*_adjusted_=2.97 × 10^−7^), further supporting independent associations in HLA class I and II loci.

### MALT versus non-MALT

Heterogeneity between the largest subtype of MZL, namely MALT and other subtypes grouped as non-MALT, was evaluated for the MZL associated SNPs (Supplementary Table 7). The effects were slightly stronger for MALT, but no evidence for substantial heterogeneity was observed (*P*_heterogeneity_≥0.05). Studies have suggested that *H. pylori* infection is a risk factor for gastric MZL^2^. An examination of SNPs previously suggested to be associated with *H. pylori* infection in independent studies^6^ did not reveal any significant association with the combined MZL or the MALT subtype in this study (Supplementary Table 8). Toll-like receptors (TLR) are considered strong candidates in mediating inflammatory immune response to pathogenic insults. A previous study reported^7^ a nominally significant association with rs4833103 in the *TLR10–TLR1–TLR6* region with MZL risk. After excluding the cases and controls in the previous report^7^, we found little additional support for this locus (MZL: *P*=0.006, OR=1.18 and MALT: *P*=0.38, OR=1.08).

### Secondary functional analyses

To gain additional insight into potential biological mechanisms, expression quantitative trait loci (eQTL) analyses were performed in two datasets consisting of lymphoblastoid cell lines (Methods). Significant associations were seen for rs2922994 and rs7750641with *HLA-B* and *HLA-C* (Supplementary Table 9) while suggestive associations (false discovery rate, FDR≤0.05) for correlated SNPs of rs2922994 (*r*^2^>0.8) in HLA class I and *RNF5* (Supplementary Table 10) were observed. No significant eQTL association was observed for rs9461741 or other correlated HLA class II SNPs. Chromatin state analysis (Methods) using ENCODE data revealed correlated SNPs of rs2922994 showed a chromatin state consistent with the prediction for an active promoter (rs3094005) or satisfied the state of a weak promoter (rs2844577) in the lymphoblastoid cell line GM12878 (Supplementary Fig. 5). GM12878 is the only lymphoblastoid cell line from which high-quality whole-genome annotation data for chromatin state is readily available. Analyses were also performed with HaploReg (Supplementary Table 11) and RegulomeDB (Supplementary Table 12) that showed overlap of the SNPs with functional motifs, suggesting plausible roles in gene regulatory processes.

## Discussion

The most statistically significant SNP associated with MZL, rs9461741, is located in HLA class II in the intron between exons 3 and 4 of the *BTNL2* gene. *BTNL2* is highly expressed in lymphoid tissues^8^ and has close homology to the B7 co-stimulatory molecules, which initiate lymphocyte activation as part of antigen presentation. Evidence is consistent with *BTNL2* acting as a negative regulator of T-cell proliferation and cytokine production^8^,^9^ and attenuating T-cell-mediated responses in the gut^10^. We were unable to statistically differentiate the effects of rs9461741 from HLA-DRB1*01:02 and, thus, our observed association could be due to *HLA-DRB1*. *HLA-DRB1* has been shown to be associated with other autoimmune diseases, including rheumatoid arthritis^11^ and selective IgA deficiency^12^. Similarly, rs2922994 is located 11 kb upstream of *HLA-B*, which is known to play a critical role in the immune system by presenting peptides derived from the endoplasmic reticulum lumen. rs7750641, a missense variant in *TCF19*, was previously associated with pleiotropic effects on blood-based phenotypes^13^ and is highly expressed in germinal center cells and up-regulated in human pro-B and pre-B cells^14^. Autoimmune diseases, such as Sjögren’s syndrome and systemic lupus erythematosus, are established risk factors for developing MZL, with the strongest associations seen between Sjögren's syndrome and the MALT subtype^15^. Of note, SNPs in *HLA-B* and the classical alleles HLA-DRB1*01:02 are strongly associated with Sjögren’s syndrome^16^, while HLA-DRB1*03 has been associated with rheumatoid arthritis^17^. The multiple independent associations in the HLA region and their localization to known functional autoimmune and B-cell genes suggest possible shared genetic effects that span both lymphoid cancers and autoimmune diseases. Chronic autoimmune stimulation leading to over-activity and defective apoptosis of B cells, and secondary inflammation events triggered by genetic and environmental factors are biological mechanisms that may contribute to the pathogenesis of MZL.

We have performed the largest GWAS of MZL to date and identified two independent SNPs within the HLA region that are robustly associated with the risk of MZL. In addition to the known diversity in etiology and pathology, there is mounting evidence of genetic heterogeneity across the NHL subtypes of lymphoma. However, the HLA region appears to be commonly associated with multiple major subtypes, such as MZL, CLL^18^, DLBCL^19^ and FL^20^,^21^,^22^,^23^. Further studies are needed to identify biological mechanisms underlying these relationships and advance our knowledge regarding their interactions with associated environmental factors that may modulate disease risks.

## Methods

### Stage 1 MZL GWAS study subjects and ethics

As part of a larger NHL GWAS initiative, we conducted a GWAS of MZL using 890 cases and 2,854 controls of European descent from 22 studies of NHL (Supplementary Table 1 and Supplementary Table 2), including nine prospective cohort studies, eight population-based case–control studies, and five clinic or hospital-based case–control studies. All studies were approved by the respective Institutional Review Boards as listed. These are ATBC:(NCI Special Studies Institutional Review Board), BCCA: UBC BC Cancer Agency Research Ethics Board, CPS-II: American Cancer Society, ELCCS: Northern and Yorkshire Research Ethics Committee, ENGELA: IRB00003888—Comite d’ Evaluation Ethique de l'Inserm IRB # 1, EPIC: Imperial College London, EpiLymph: International Agency for Research on Cancer, HPFS: Harvard School of Public Health (HSPH) Institutional Review Board, Iowa-Mayo SPORE: University of Iowa Institutional Review Board, Italian GxE: Comitato Etico Azienda Ospedaliero Universitaria di Cagliari, Mayo Clinic Case–Control: Mayo Clinic Institutional Review Board, MCCS: Cancer Council Victoria’s Human Research Ethics Committee, MD Anderson: University of Texas MD Anderson Cancer Center Institutional Review Board, MSKCC: Memorial Sloan-Kettering Cancer Center Institutional Review Board, NCI-SEER (NCI Special Studies Institutional Review Board), NHS: Partners Human Research Committee, Brigham and Women's Hospital, NSW: NSW Cancer Council Ethics Committee, NYU-WHS: New York University School of Medicine Institutional Review Board, PLCO: (NCI Special Studies Institutional Review Board), SCALE: Scientific Ethics Committee for the Capital Region of Denmark, SCALE: Regional Ethical Review Board in Stockholm (Section 4) IRB#5, UCSF2: University of California San Francisco Committee on Human Research, WHI: Fred Hutchinson Cancer Research Center, Yale: Human Investigation Committee, Yale University School of Medicine. Informed consent was obtained from all participants.

Cases were ascertained from cancer registries, clinics or hospitals or through self-report verified by medical and pathology reports. To determine the NHL subtype, phenotype data for all NHL cases were reviewed centrally at the International Lymphoma Epidemiology Consortium (InterLymph) Data Coordinating Center and harmonized using the hierarchical classification proposed by the InterLymph Pathology Working Group^24^,^25^ based on the World Health Organization (WHO) classification^26^.

### Genotyping and quality control

All MZL cases with sufficient DNA (*n*=890) and a subset of controls (*n*=2,854) frequency matched by age, sex and study to the entire group of NHL cases, along with 4% quality control duplicates, were genotyped on the Illumina OmniExpress at the NCI Core Genotyping Resource (CGR). Genotypes were called using Illumina GenomeStudio software, and quality control duplicates showed >99% concordance. Monomorphic SNPs and SNPs with a call rate of <95% were excluded. Samples with a call rate of ≤93%, mean heterozygosity <0.25 or >0.33 based on the autosomal SNPs or gender discordance (>5% heterozygosity on the X chromosome for males and <20% heterozygosity on the X chromosome for females) were excluded. Furthermore, unexpected duplicates (>99.9% concordance) and first-degree relatives based on identity by descent sharing with Pi-hat >0.40 were excluded. Ancestry was assessed using the Genotyping Library and Utilities (GLU- http://code.google.com/p/glu-genetics/) struct.admix module based on the method by Pritchard *et al.*^27^ and participants with <80% European ancestry were excluded (Supplementary Fig. 1). After exclusions, 825 cases and 2,685 controls remained (Supplementary Table 2). Genotype data previously generated on the Illumina Omni2.5 from an additional 3,536 controls from three of the 22 studies (ATBC, CPS-II and PLCO) were also included^3^, resulting in a total of 825 cases and 6,221 controls for the stage 1 analysis (Supplementary Table 3). Of these additional 3,536 controls, 703 (~235 from each study) were selected to be representative of their cohort and cancer free^3^, while the remainder were cancer-free controls from an unpublished study of prostate cancer in the PLCO. SNPs with call rate <95%, with Hardy–Weinberg equilibrium *P*<1 × 10^−6^, or with a MAF <1% were excluded from analysis, leaving 611,856 SNPs for analysis. To evaluate population substructure, a principal components analysis was performed using the Genotyping Library and Utilities (GLU), version 1.0, struct.pca module, which is similar to EIGENSTRAT^28^ - http://genepath.med.harvard.edu/~reich/Software.htm. Plots of the first five principal components are shown in Supplementary Fig. 2. Genomic inflation factor was computed prior (*λ*=1.014) and after removal of SNPs in the HLA loci (*λ*=1.010). Association testing was conducted assuming a log-additive genetic model, adjusting for age, sex and three significant principal components. All data analyses and management were conducted using GLU.

### Imputation of variants

To more comprehensively evaluate the genome for SNPs associated with MZL, SNPs in the stage 1 discovery GWAS were imputed using IMPUTE2 (ref. 29)- http://mathgen.stats.ox.ac.uk/impute/impute_v2.html and the 1000 Genomes Project (1kGP- http://www.1000genomes.org/) version 3 data^29^,^30^. SNPs with a MAF <1% or information quality score (info) <0.3 were excluded from analysis, leaving 8,478,065 SNPs for association testing. Association testing on the imputed data was conducted using SNPTEST^31^— https://mathgen.stats.ox.ac.uk/genetics_software/snptest/snptest.html version 2, assuming dosages for the genotypes and adjusting for age, sex and three significant principal components. In a null model for MZL risk, the three eigenvectors EV1, EV3 and EV8 were nominally associated with MZL risk and hence were included to account for potential population stratification. Heterogeneity between MZL subtypes was assessed using a case–case comparison, adjusting for age, sex and significant principal components.

### Stage 2 replication of SNPs from the GWAS

After ranking the SNPs by *P* value and LD filtering (*r*^*2*^<0.05), 10 SNPs from the most promising loci identified from stage 1 after imputation with *P*<7.5 × 10^−6^ were taken forward for *de novo* replication in an additional 456 cases and 906 controls (Supplementary Tables 1 and 4). Wherever possible, we selected either the best directly genotyped SNP or the most significant imputed SNP for the locus. Only imputed SNPs with an information score >0.8 were considered for replication. Only SNPs with MAF >1% were selected for replication, and no SNPs were taken forward for replication in regions where they appeared as singletons or obvious artifacts. For the HLA region, we selected one additional SNP (rs7750641) that was highly correlated with rs2922994 for additional confirmation. Genotyping was conducted using custom TaqMan genotyping assays (Applied Biosystems) validated at the NCI Core Genotyping Resource. Genotyping was done at four centres. HapMap control samples genotyped across two centres yielded 100% concordance as did blind duplicates (~5% of total samples). Due to the small number of samples, the MD Anderson, Mayo and NCI replication studies were pooled together for association testing; however, MSKCC samples were analysed separately to account for the available information on Ashkenazi ancestry. Association results were adjusted for age and gender and study site in the pooled analysis. The results from the stage 1 and stage 2 studies were then combined using a fixed effect meta-analysis method with inverse variance weighting based on the estimates and s.e. from each study. Heterogeneity in the effect estimates across studies was assessed using Cochran’s Q statistic and estimating the *I*^2^ statistic. For all SNPs that reached genome-wide significance in Table 1, no substantial heterogeneity was observed among the studies (*P*_heterogeneity_≥0.1 for all SNPs, Supplementary Table 4).

### Technical validation of imputed SNPs

Genotyping was conducted using custom TaqMan genotyping assays (Applied Biosystems) at the NCI Cancer Genomics Research Laboratory on a set of 470 individuals included in the stage 1 MZL GWAS. The allelic dosage *r*^2^ was calculated between the imputed genotypes and the technical validation done using assayed genotypes which showed that both SNPs were imputed with high accuracy (INFO ≥0.99) and a high correlation (*r*^2^≥0.99) between dosage imputation and genotypes obtained by Taqman assays.

### HLA imputation and analysis

To determine if specific coding variants within HLA genes contributed to the diverse association signals, we imputed the classical HLA alleles (*A*, *B*, *C*, *DQA1*, *DQB1*, *DRB1*) and coding variants across the HLA region (chr6:20–40 Mb) using SNP2HLA^5^- http://www.broadinstitute.org/mpg/snp2hla/. The imputation was based on a reference panel from the Type 1 Diabetes Genetics Consortium (T1DGC) consisting of genotype data from 5,225 individuals of European descent who were typed for *HLA-A*, *B*, *C*, *DRB1*, *DQA1*, *DQB1*, *DPB1*, *DPA1* 4-digit alleles. Imputation accuracy of HLA alleles was assessed by comparing HLA alleles to the HLA sequencing data on a subset of samples from the NCI^32^. The concordance rates obtained were 97.32, 98.5, 98.14 and 97.49% for *HLA-A*, *B*, *C* and *DRB1*, respectively, in the NCI GWAS suggesting robust performance of the imputation method. Due to the limited number of SNPs (7,253) in the T1DGC reference set, imputation of HLA SNPs was conducted with IMPUTE2 and the 1kGP reference set as described above. A total of 68,488 SNPs, 201 classical HLA alleles (two- and four-digit resolution) and 1,038 AA markers including 103 AA positions that were ‘multi-allelic’ with three to six different residues present at each position, were successfully imputed (info score >0.3 for SNPs or *r*^*2*^>0.3 for alleles and AAs) and available for downstream analysis. Multi-allelic markers were analysed as binary markers (for example, allele present or absent) and a meta-analysis was conducted where we tested SNPs, HLA alleles and AAs across the HLA region for association with MZL using PLINK^33^ or SNPTEST^31^ as described above.

### eQTL analysis

We conducted an eQTL analysis using two independent datasets: childhood asthma^34^ and HapMap^35^. As described previously^34^ for the childhood asthma data set^35^, peripheral blood lymphocytes were transformed into lymphoblastoid cell lines for 830 parents and offspring from 206 families of European ancestry. Data from 405 children were used for the analysis as follows: using extracted RNA, gene expression was assessed with the Affymetrix HG-U133 Plus 2.0 chip. Genotyping was conducted using the Illumina Human-1 Beadchip and Illumina HumanHap300K Beadchip, and imputation performed using data from 1kGP. All SNPs selected for replication were tested for *cis* associations (defined as gene transcripts within 1 Mb), assuming an additive genetic model, adjusting for non-genetic effects in the gene expression value. Association testing was conducted using a variance component-based score test^36^ in MERLIN^37^, which accounts for the correlation between siblings. To gain insight into the relative importance of associations with our SNPs compared with other SNPs in the region, we also conducted conditional analyses, in which both the MZL SNP and the most significant SNP for the particular gene transcript (that is, peak SNP) were included in the same model. Only *cis* associations that reached *P*<6.8 × 10^−5^, which corresponds to a FDR of 1% are reported (Supplementary Table 9).

The HapMap data set consisted of a publicly available RNAseq data set^35^ from transformed lymphoblastoid cell lines from 41 CEPH Utah residents with ancestry from northern and western Europe (HapMap-CEU) samples available from the Gene Expression Omnibus repository ( http://www.ncbi.nlm.nih.gov/geo) under accession number GSE16921. In this data set, we examined the association between the two reported SNPs in the HLA region, rs2922994 and rs9461741, as well as all SNPs in LD (*r*^2^>0.8 in HapMap-CEU release 28) and expression levels of probes within 1 Mb of the SNPs. As rs9461741 was not genotyped in HapMap, we selected rs7742033 as a proxy as it was the strongest linked SNP available in HapMap (*r*^2^=0.49 in 1kGP-CEU). Genotyping data for these HapMap-CEU individuals were directly downloaded from HapMap ( www.hapmap.org). Correlation between expression and genotype for each SNP-probe pair was tested using the Spearman’s rank correlation test with *t*-distribution approximation and estimated with respect to the minor allele in HapMap-CEU. *P* values were adjusted using the Benjamini–Hochberg FDR correction and eQTLs were considered significant at an FDR<0.05 (Supplementary Table 10).

### Bioinformatics ENCODE and chromatin state dynamics

To assess chromatin state dynamics, we used Chromos^38^, which has precomputed data from ENCODE on nine cell types using Chip-Seq experiments^39^. These consist of B-lymphoblastoid cells (GM12878), hepatocellular carcinoma cells (HepG2), embryonic stem cells, erythrocytic leukaemia cells (hK562), umbilical vein endothelial cells, skeletal muscle myoblasts, normal lung fibroblasts, normal epidermal keratinocytes and mammary epithelial cells. These precomputed data have genome-segmentation performed using a multivariate hidden Markov model to reduce the combinatorial space to a set of interpretable chromatin states. The output from Chromos lists data into 15 chromatin states corresponding to repressed, poised and active promoters, strong and weak enhancers, putative insulators, transcribed regions and large-scale repressed and inactive domains (Supplementary Fig. 5).

## Additional information

**How to cite this article:** Vijai, J. *et al.* A genome-wide association study of marginal zone lymphoma shows association to the HLA region. *Nat. Commun.* 6:5751 doi: 10.1038/ncomms6751 (2015).

## Supplementary Material

Supplementary Information Supplementary Figures 1-5, Supplementary Tables 1-12

## Figures and Tables

**Table 1:** Association results for two new independent SNPs with MZL in a two-stage GWAS.

**Chr**	**Nearest gene(s)**	**SNP**	**Position** *	**Risk allele** †	**Other allele**	**RAF** ‡	**Stage**	**No. of cases/no. of controls**	**OR**	**95% CI**	***P*** **value**§	***P***_**heterogeneity**_	***I*** ^2^
6p21.32	* BTNL2 *	rs9461741	32370587	C	G	0.018	Stage 1	824/6,220	2.40	(1.74–3.31)	9.11E-08		
						0.030	Stage 2	453/877	3.06	(2.10–4.46)	5.24E-09		
							Combined	1,277/7,097	2.66	(2.08–3.39)	3.95E-15	0.216	34.69
6p21.33	* HLA-B *	rs2922994	31335901	G	A	0.113	Stage 1	825/6,221	1.74	(1.43–2.12)	2.89E-08		
						0.094	Stage 2	405/832	1.43	(1.08–1.90)	0.01		
							Combined	1,230/7,053	1.64	(1.39–1.92)	2.43E-09	0.507	0

CI, confidence interval; GWAS, genome-wide association study; MZL, marginal zone lymphoma; OR, odds ratio; RAF, risk allele frequency; SNP, single-nucleotide polymorphism.

^*^Position according to human reference NCBI37/hg19.

^†^Allele associated with an increased risk of MZL.

^‡^Risk allele frequency in controls.

^§^For stage 1 and 2, *P* values were generated by using logistic regression. For the combined stage, the odds ratio and *P* values were generated using a fixed effects model. Heterogeneity in the effect estimates was assessed using Cochran’s *Q* statistic and estimating the *I*^*2*^ statistic.
