# Supplementary material for: A genome-wide association study of marginal zone lymphoma shows association to the HLA region
Source: Nat Commun. 2015 Jan 8;6:5751. doi: 10.1038/ncomms6751 (PMC4287989; doi:10.1038/ncomms6751)
Supplement: Supplementary Information — Supplementary Figures 1-5, Supplementary Tables 1-12 [file ncomms6751-s1.pdf]

SUPPLEMENTARY FIGURES

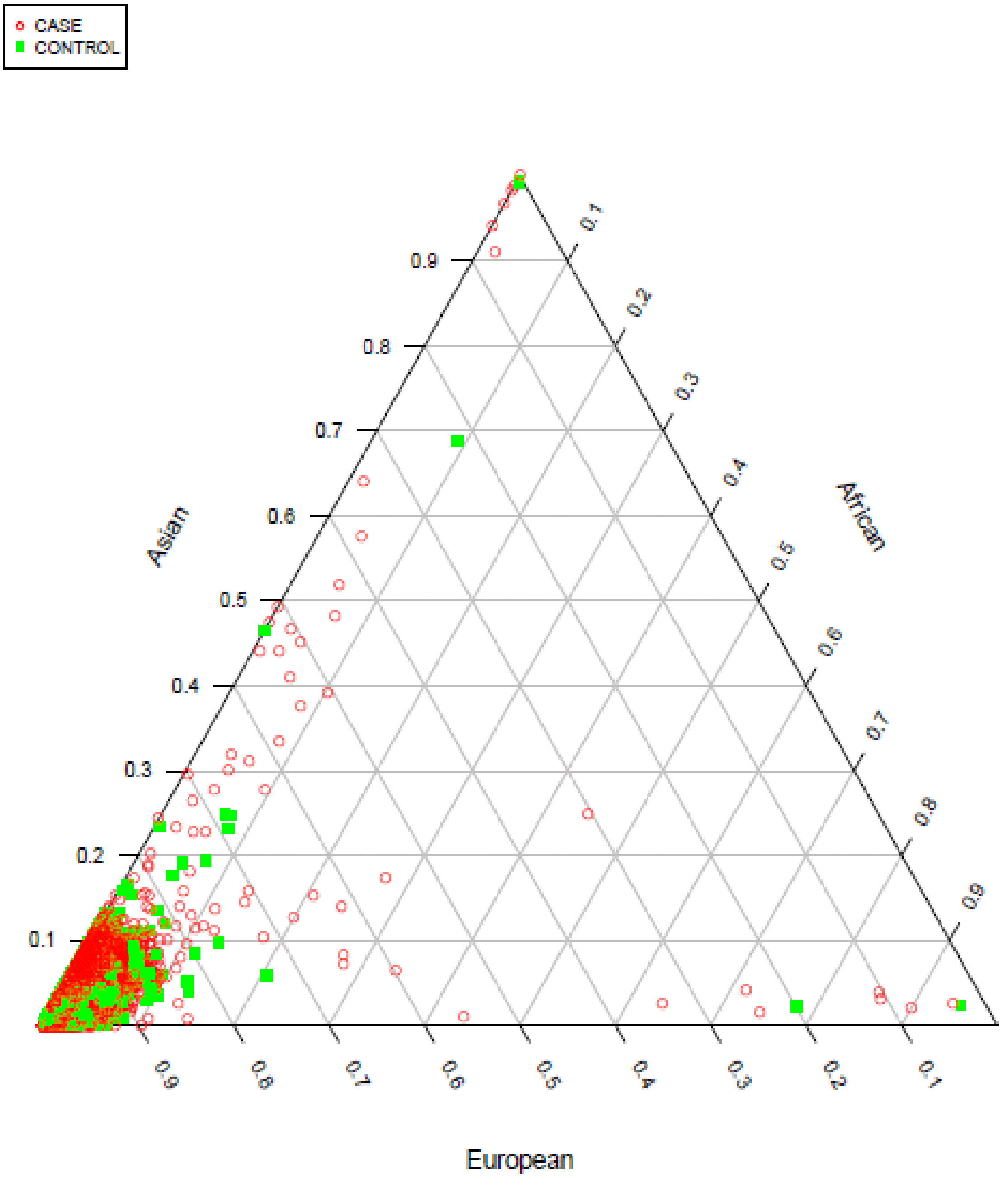

Supplementary Figure 1. Plot of estimated admixture for individuals in MZL GWAS (stage 1). For details, see the Methods. Individuals with <80% European ancestry were excluded.

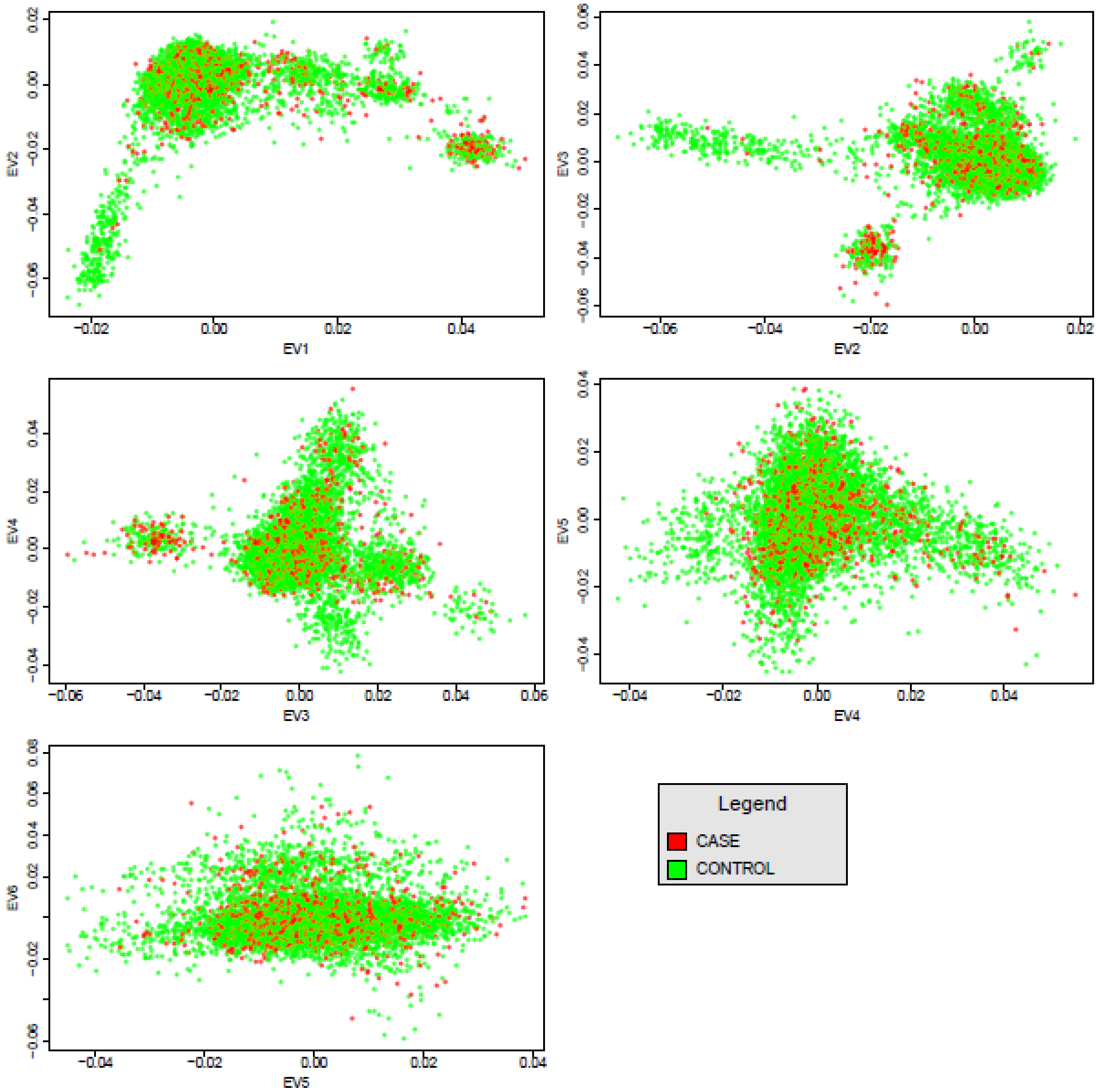

Supplementary Figure 2. Plot of top eigenvectors from MZL GWAS (stage 1) data based on principal components analysis. For details, see Methods.

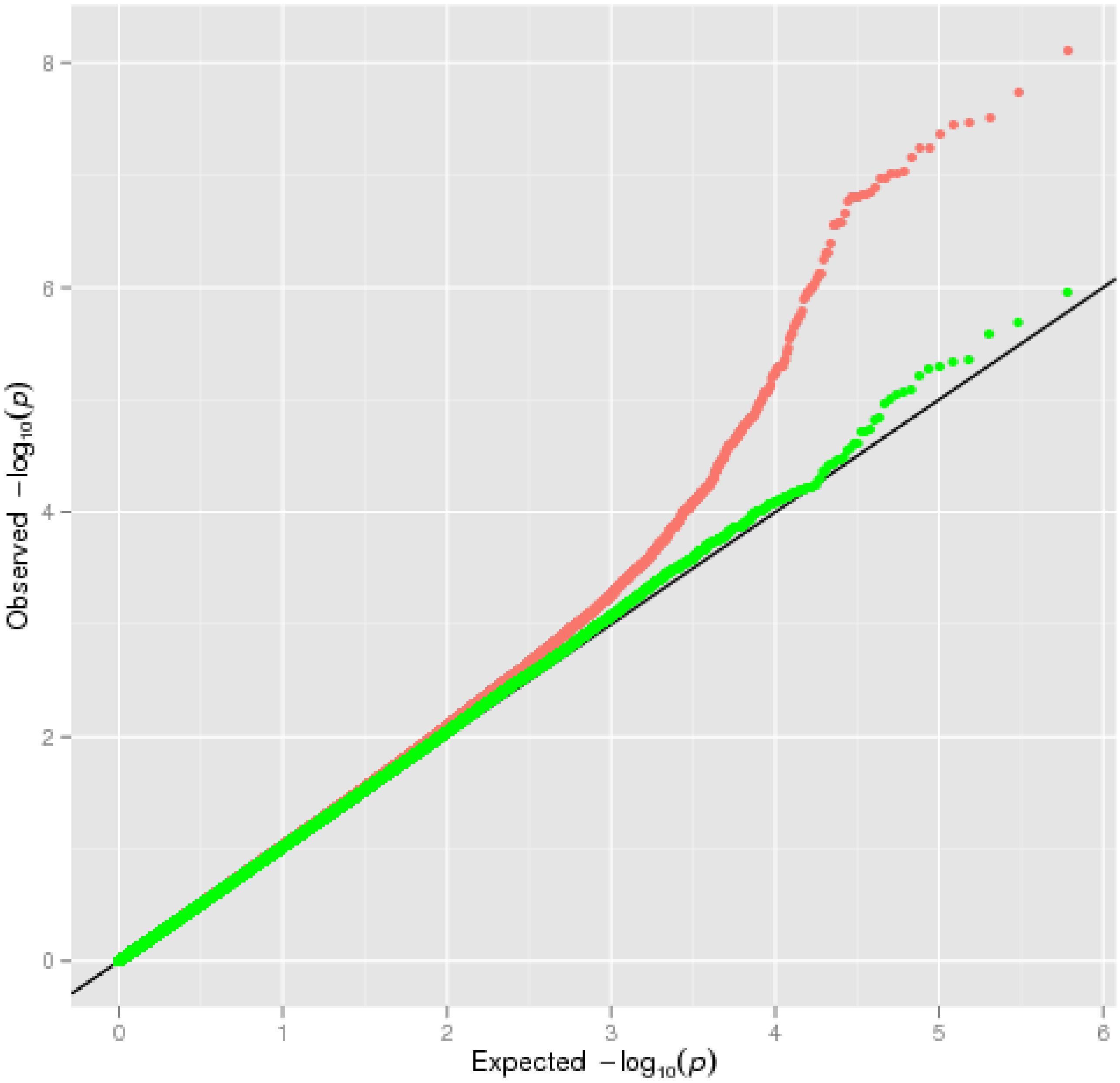

Supplementary Figure 3. Quantile-quantile (Q-Q) plot of the association results for MZL from stage 1 (NHL-GWAS) (red) and after removing SNPs located in the HLA region (green)

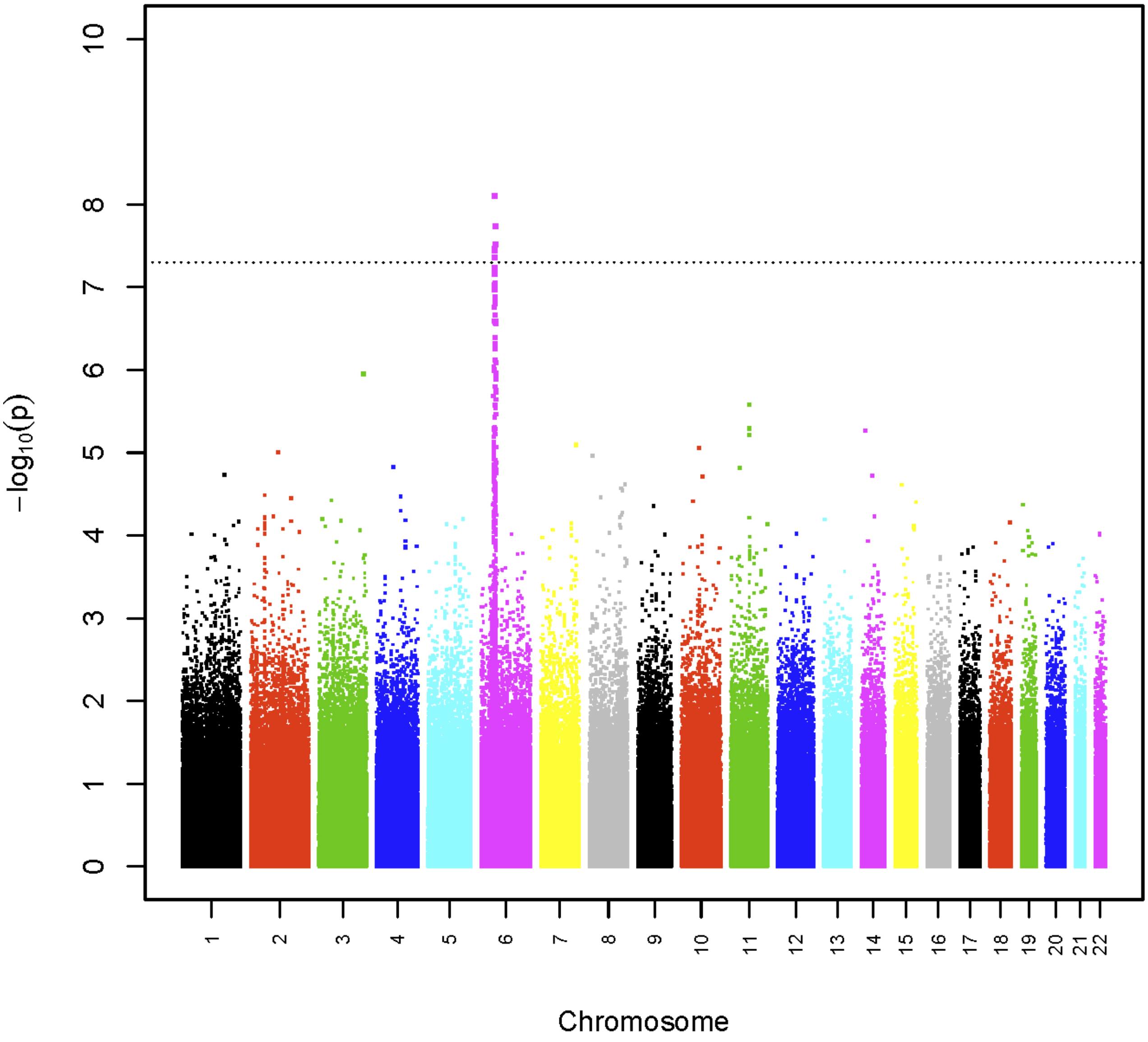

Supplementary Figure 4. Manhattan plot of the MZL GWAS (Stage 1) showing chromosomes on X axis and the –log$_{10}$ of *P*-values on Y axis

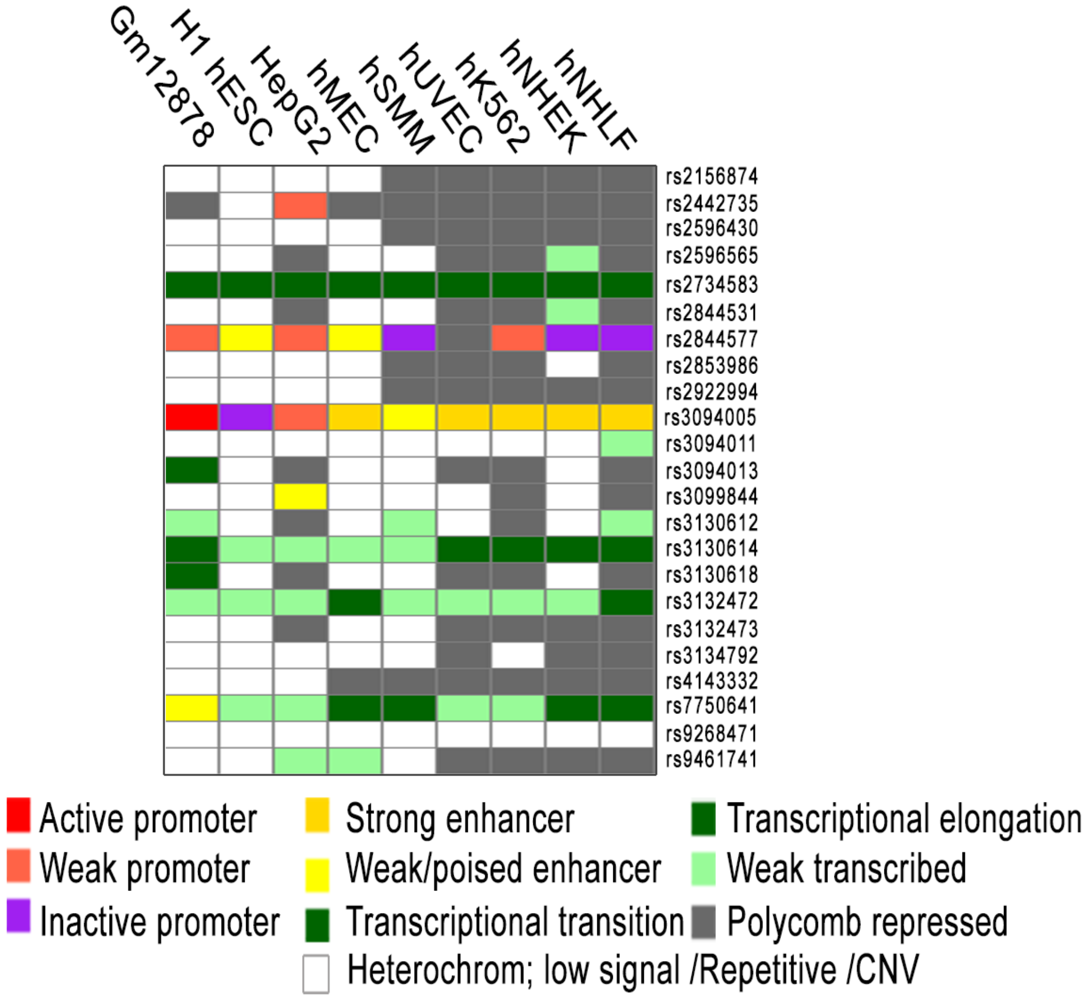

Supplementary Figure 5. Chromatin states affected by MZL GWAS SNPs and their correlated SNPs (r^2=1) from 1000 Genomes data

SUPPLEMENTARY TABLES

Supplementary Table 1. Description and design of studies included in stage 1 and stage 2 comprising 1397 cases and 7782 controls

Study Name	Study Abbreviation	No. MZL Cases[a]	No. Controls[a]	Design, location	Source of cases	Source of controls	Study Reference
**Stage 1 - NHL-GWAS**							
**Cohort Studies**							
Alpha-Tocopherol, Beta-Carotene Lung Cancer Prevention Study	ATBC	1	240[b]	Nested case-control, Finland	Identified through linkage to the Finnish Cancer Registry	Cohort participants without a diagnosis of cancer	**[PMID: 8205268]** The alpha-tocopherol, beta-carotene lung cancer prevention study: design, methods, participant characteristics, and compliance. The ATBC Cancer Prevention Study Group. Ann Epidemiol. 1994 Jan;4(1):1-10.
American Cancer Society Cancer Prevention Study-II Nutrition Cohort	CPS-II	56	220[b]	Nested case-control, USA	Self-report through biannual questionnaires (starting in 1997). Verified by medical records or linkage to state cancer registry.	Cohort participants alive at time of case diagnosis without cancer	**[PMID:11900235]** Calle EE. et al. The American Cancer Society Cancer Prevention Study II Nutrition Cohort: rationale, study design, and baseline characteristics. Cancer. Cancer 2002;94:2490-501.
European Prospective Investigation into Cancer, Chronic Diseases, Nutrition and Lifestyles	EPIC	13	773	Nested case-control, multiple European countries	Cases identified through population cancer registries in seven of the participating countries (Denmark, Italy, The Netherlands, Norway, Spain, Sweden and the UK) and through a combination of methods including health insurance records, cancer and pathology registries, and by active follow-up through study subjects and their next-of-kin in three countries (France, Germany and Greece).	Cohort participants matched by age, sex and study center who were alive and cancer-free at the time of diagnosis of the corresponding case	**[PMID:9126529]**Riboli E. et al. The EPIC Project: rationale and study design. European Prospective Investigation into Cancer and Nutrition. Int J Epidemiol. Int J of Epidemiol 1997;26(1):S6-14. **[PMID:12639222]** Riboli E. et al. European Prospective Investigation into Cancer and Nutrition (EPIC): study populations and data collection. Public Health Nutr. Public Health Nutr 2002;5(6B):1113-24.

Health Professionals Follow-up Study	HPFS	5	86	Nested case-control, USA	Self-report through bi-annual questionnaires. Verified by medical records and pathology report	Cohort participants alive at time of case diagnosis without cancer, matched on date of birth, ethnicity, date and time of day of blood collection, and fasting status	**[PMID: 1678444]** Rimm E. et al. Prospective study of alcohol consumption and risk of coronary disease in men. Lancet. Lancet 1991;338:464-8.
The Melbourne Collaborative Cohort Study	MCCS	9	246	Nested case-control, Australia	Incident cases ascertained through national cancer registries	Controls were unaffected cohort participants	**[PMID: 12484128]** Giles GG. et al. The Melbourne Collaborative Cohort Study. IARC Sci Publ. IARC Sci Publ 2002;156:69-70.
Nurses' Health Study	NHS	12	90	Nested case-control, USA	Self-report through bi-annual questionnaires. Verified by medical records and pathology report	Cohort participants alive at time of case diagnosis without cancer, matched on date of birth, ethnicity, date and time of day of blood collection, and fasting status	**[PMID: 15864280]** Colditz GA. et al. The Nurses' Health Study: lifestyle and health among women. Nat Rev Cancer. Nat Rev Cancer 2005;5:388-96. **[PMID: 7658481]** Hankinson SE. et al. Alcohol, height and adiposity in relation to estrogen and prolactin levels in postmenopausal women. J Natl Cancer Inst. J Natl Cancer Inst 1995;87:1297-302.
New York University Women's Health Study	NYU-WHS	6	56	Nested case-control, USA	Self-report through questionnaires every 2-4 years, confirmed by medical and pathology records; and linkages to tumor registries of NY, NJ and Florida and NDI	Cohort participants selected by incidence density sampling (alive and free of cancer at time of case diagnosis	**[PMID: 7707406]** Toniolo P. et al. A prospective study of endogenous estrogens and breast cancer in postmenopausal women. J Natl Cancer Inst. J Natl Cancer Inst 1995; 87:190-7. **[PMID: 20373009]** Gu Y. et al. Circulating cytokines and risk of B-cell non-Hodgkin lymphoma: a prospective study. Cancer Causes Control. Cancer Causes Control 2010; 21(8):1323-33.
Prostate, Lung, Colorectal, and Ovarian Cancer Screening Trial	PLCO	28	3076[b]	Nested case-control, USA	Self-report through annual questionnaires. Verified by medical records and pathology report	Cohort participants alive at time of case diagnosis without cancer diagnosis	**[PMID: 20494998]** Troy JD, et al. Associations between anthropometry, cigarette smoking, alcohol consumption, and non-Hodgkin lymphoma in the Prostate, Lung, Colorectal, and Ovarian Cancer Screening Trial. Am J Epidemiol. Am J Epidemiol 2010;171:1270-81. **[PMID: 16054167]** Hayes RB et al. Methods for etiologic and early marker investigations in the PLCO trial. Mutat Res. Mutat Res 2005;592:147-54.

Women's Health Initiative	WHI	91	395	Nested case-control, USA	Self-report through semi-annual clinic visits or annual contact. Verified through medical records	Cohort participants without a diagnosis of cancer	**[PMID: 14575938]** Anderson GL, et al. Implementation of the Women's Health Initiative study design. Ann Epidemiol. 2003 Oct;13(9 Suppl):S5-17.
**Population-based case-control studies**							
British Columbia Non-Hodgkin Lymphoma Study	BCCA	41	390	Population-based case-control study, Canada	First primary NHL diagnosis from Vancouver and Victoria metropolitan areas identified through the BC Cancer Registry (excluding HIV-infected and post-transplant cases)	Controls from the same areas, matched on area, age, and sex ascertained from the British Columbia Health Insurance files	**[PMID:17722095]** Spinelli JJ. et al. Organochlorines and risk of non-Hodgkin lymphoma. Int J Cancer. Int J Cancer 2007; 121(12):2767-75.
Epidemiology & Genetics Unit Lymphoma Case-Control study	ELCCS	0	461	Population-based case-control study, UK	Cases were patients aged between 18-69 residing in predefined geographic areas and newly diagnosed with NHL between 1998 and 2003. Diagnoses were pathologically confirmed and coded to the WHO Classification for Oncology	For each case, one age- and sex- matched control was randomly selected from population based General practice registers	**[PMID: 15456990]** Willett EV. et al. Tobacco and alcohol consumption and the risk of non-Hodgkin lymphoma. Cancer Causes Control. Cancer Causes Control 2004;15:771-80. **[PMID: 19736055]** Worrillow L. et al. Polymorphisms in the nucleotide excision repair gene ERCC2/XPD and risk of non-Hodgkin lymphoma. Cancer Epidemiol. Cancer Epidemiol 2009;33(3-4):257-60. **[PMID 20832384]** Crouch S. et al. Illness patterns prior to diagnosis of lymphoma: analysis of UK medical records. Cancer Epidemiol. Cancer Epidemiol 2001;35(2):145-50.
Multicenter Italian study on gene-environment interactions in lymphoma etiology: translational aspects	Italian GxE	8	54	Population-based case-control study, Italy	First primary NHL diagnosis identified in the Hematology Departments of the participating centres	Cohort participants alive at time of case diagnosis without cancer	
National Cancer Institute-Surveillance, Epidemiology, and End Results Interdisciplinary Case-Control Study of Non-Hodgkin's Lymphoma	NCI-SEER	65	689	Population-based case-control study, USA	First primary NHL diagnosis identified through 4 SEER registries (excluding HIV-infected cases)	Controls from the same areas, matched on area, age, and race ascertained though random digit dialing (<64 years of age) and CMMS files (≥65 years of age)	**[PMID: 15342441]** Chatterjee N. et al. Risk of non-Hodgkin's lymphoma and family history of lymphatic, hematology, and other cancers. CEBP. CEBP 2004;13:1415-21. **[PMID: 17018637]** Wang SS. et al. Common genetic variants in proinflammatory and other immunoregulatory genes and risk for non-Hodgkin lymphoma. Cancer Res. Cancer Res 2006;66(19):9771-80.

NSW non-Hodgkin lymphoma study	NSW	39	397	Population-based case-control study, Australia and New Zealand	Incident NHL diagnosis identified through NSW or ACT cancer registry (excluding HIV-infected cases and transplant recipients)	Controls randombly selected from electoral rolls, matched on age, sex and State of residence at diagnosis	**[PMID: 15095310]** Hughes AM, et al. Pigmentary characteristics, sun sensitivity and non-Hodgkin lymphoma. IJC. IJC 2004;110:429-34.
Scandinavian Lymphoma Etiology Study	SCALE	64	301	Population-based case-control study, Scandinavia	Patients with incident primary NHL diagnosed through rapid case-ascertainment network in Sweden and Denmark	Frequency matched (age in 10 year intervals, sex and country) population controls prospectively identified every 6 months in nationwide population registers (incidence density sampling).	**[PMID: 15687363]** Smedby KE. et al. Ultraviolet radiation exposure and risk of malignant lymphomas. J Natl Cancer Inst. J Natl Cancer Inst 2005;97(3)199-209.
Molecular Epidemiology of non-Hodgkin lymphoma	UCSF2	94	10	Population-based case-control study, USA	RCA/SEER Incident NHL diagnosis for patients diagnosed in hospitals in 6 San Francisco Bay Area Counties and who were residents of the Bay Area at the time of diagnosis	Controls ascertained through RDD were frequency matched to cases on age in 5-year groups, sex and county of residence; Random sampling of CMS lists for person residing in the same 6 Bay Area counties were used to supplement recruitment of controls aged 65+	[**PMID: 20639881**] Skibola CF. et al. Genetic variants at 6p21.33 are associated with susceptibility to follicular lymphoma. Nat Genet. Nat Genet 2009;41(8):873-5. [**PMID: 22697504**] Mikhak B. et al. Intake of vitamins d and a and calcium and risk of non-Hodgkin lymphoma: San Francisco Bay Area population-based case-control study. Nutr Cancer. Nutr Cancer 2012;64(5):674-84.
Population-based case-control study in Connecticut women	Yale	28	504	Population-based case-control study, USA	First primary NHL diagnosis identified through the Rapid Case Shared Resources from all the hospitals in Connecticut	Population-based controls through random digit dialing for cases <65 years and Medicare files for ≥65 years	**[PMID: 19822571]** Zhang Y et al. Genetic variations in xenobiotic metabolic pathway genes, personal hair dye use and risk of non-Hodgkin lymphoma. Am J Epidemiol. Am J Epidemiol 2009;170(10):1222-30.
**Clinic or hospital-based or mixed case-control studies**							
Environmental and genetic risks factors study in adult lymphoma	ENGELA	8	278	Hospital-based case-control study, France	Recent diagnosis of a NHL as per the WHO classification (ICD-O-3) / Cases with AIDS or on immunosuppressant drugs were not eligible. Path reports for 100%, slides review for selected NHL	Hospitalized in the same hospitals as the cases, for any reason except cancer, an accident or a disease directly related to the subject’s occupation, smoking, or alcohol consumption. HIV negative.	**[PMID: 18781390]** Monnereau A. et al. Cigarette smoking, alcohol drinking, and risk of lymphoid neoplasms: results of a French case-control study. Cancer Causes Control. Cancer Causes Control 2008;19(10):1147-60.

Epilymph case-control study in six European countries	EpiLymph	71	1172	Multicenter case-control study, hospital-based and population-based, Europe	First primary lymphoma diagnosis (according to the 2001 WHO classification of lymphoma	Controls from Germany and Italy were randomly selected by sampling from the general population, matched to cases on gender, 5-year age-group, and residence area. The rest of the centers used matched hospital controls, with eligibility criteria limited to diagnoses other than cancer, infectious diseases and immunodeficient diseases	**[PMID:16557575]** Besson H. et al. Tobacco smoking, alcohol drinking and non-Hodgkin's lymphoma: A European multicenter case-control study (Epilymph). Int J Cancer. Int J Cancer 2006;119:901-8.
Iowa-Mayo SPORE Molecular Epidemiology Resource	Iowa-Mayo SPORE	113	0	Clinic-based case registry, USA	Consecutive patients with newly diagnosed, histologically-confirmed non-Hodgkin lymphoma (excluding HIV-infected cases) who were residents of US	N/A	**[PMCID:PMC2953973]** Drake MT. et al. Vitamin D insufficiency and prognosis in non-Hodgkin's lymphoma. J Clin Oncol. J Clin Oncol 2010;28:4191-8.
Mayo Clinic Case-Control Study of NHL and CLL	Mayo Case-Control	78	911	Clinic-based case-control study, USA	Consecutive patients with newly diagnosed, histologically-confirmed non-Hodgkin lymphoma (excluding HIV-infected cases) who were residents of Minnesota, Iowa or Wisconsin	Controls were selected from patients seen in the general medicine clinics at Mayo with a pre-scheduled general medical examination, frequency on age, sex, and geographic region	**[PMCID:PMC3110384]** Cerhan JR. et al. Design and validity of a clinic-based case-control study on the molecular epidemiology of lymphoma. Int J Mol Epidemiol Genet. Int J Mol Epidemiol Genet 2011;2(2):95-113.
Memorial-Sloan Kettering Lymphoproliferative disorders Study	MSKCC	67	9	Hospital-based case-study and NYCP controls, USA	Hospital clinic based ascertainment in a tertiary referral center	NYCP controls from same geographic area	**[PMID: 12691155]** Yossepowitch O. et al. BRCA1 and BRCA2 germline mutations in lymphoma patients. Leuk Lymphoma. Leuk Lymphoma 2003;44(1):127-31.
**Stage 2 - Replication studies**							
Iowa-Mayo SPORE Molecular Epidemiology Resource	Iowa-Mayo SPORE	60	0	Clinic-based case registry, USA	Consecutive patients with newly diagnosed, histologically-confirmed non-Hodgkin lymphoma (excluding HIV-infected cases) who were residents of US	N/A	**[PMCID:PMC2953973]** Drake MT. et al. Vitamin D insufficiency and prognosis in non-Hodgkin's lymphoma. J Clin Oncol. J Clin Oncol 2010;28:4191-8.
Mayo Clinic Case-Control Study of NHL	Mayo Case-Control	55	386	Clinic-based case-control study, USA	Consecutive patients with newly diagnosed, histologically-confirmed non-Hodgkin lymphoma (excluding HIV-infected cases) who were residents of	Controls were selected from patients seen in the general medicine clinics at Mayo with a pre-scheduled general medical examination, frequency on	**[PMCID:PMC3110384]** Cerhan JR. et al. Design and validity of a clinic-based case-control study on the molecular epidemiology of lymphoma. Int J Mol Epidemiol Genet. Int J Mol Epidemiol Genet

					Minnesota, Iowa or Wisconsin	age, sex, and geographic region	2011;2(2):95-113.
MD Anderson lymphoma case-control study	MD Anderson	74	76	Case-control, USA	MD Anderson Cancer Center	Kelsey Seybold Clinics	
Memorial-Sloan Kettering Lymphoproliferative disorders Study	MSKCC	223	378	Hospital-based case-study and NYCP controls, USA	Hospital clinic based ascertainment in a tertiary referral center	NYCP controls from same geographic area	**[PMID: 23349640]** Susceptibility loci associated with specific and shared subtypes of lymphoid malignancies. PLoS Genet. 2013;9(1):e1003220.
NCI Replication Study	NCI Rep	88	120	Mixed study of population and hospital-based cases and controls	MZL cases from the stage 1 studies that did not have sufficient DNA for scanning or failed in scanning due to low completion.	Subset of controls from the stage 1 studies that were not scanned or failed scanning due to low completion.	

[a]Number of cases and controls with DNA available.
[b]Controls scanned previously on the Illumina Omni2.5.

Supplementary Table 2. Stage 1 subjects genotyped, quality control exclusions, and subjects included in the analysis

			Exclusions												
	Genotyped Subjects		High missing rate		Gender discordance		Unexpected duplicates		Non CEU		Previously scanned		Final subjects included in the analysis		
**Study**	**Cases**	**Controls**	**Cases**	**Controls**	**Cases**	**Controls**	**Cases**	**Controls**	**Cases**	**Controls**	**Cases**	**Controls**	**Cases**	**Controls**	**Total**
**Cohort studies**	-	-	-	-	-	-	-	-	-	-	-	-	-	-	-
ATBC	1	-	-	-	-	-	-	-	-	-	-	240	1	240	241
CPS-II	56	-	4	-	-	-	-	-	-	-	-	220	52	220	272
EPIC	10	275	2	9	-	1	-	-	-	-	-	-	8	265	273
HPFS	5	86	-	0	-	-	-	-	-	1	-	-	5	85	90
MCCS	9	76	1	1	-	-	-	-	-		-	-	8	75	83
NHS	12	90	-	2	-	-	-	-	-	-	-	-	12	88	100
NYU-WHS	6	56	-	3	-	-	-	-	-	-	-	-	6	53	59
PLCO	28	-	-	-	-	-	2	-	-	-	-	3076	26	3076	3102
WHI	91	250	5	19	-	-	1	-	1	3	-	-	84	228	312
**Subtotal**	218	833	12	34	-	1	3	-	1	4	-	3536	202	4330	4532
**Population-based case-control studies**			-	-	-	-	-	-	-	-	-	-	-	-	-
BCCA	41	110	1	1	-	-	-	-	-	-	-	-	40	109	149
ELCCS	-	251	-	5	-	1	-	-	-	-	-	-	-	245	245
Italian GxE	9	54	3	8	-	1	-	-	-	-	-	-	6	45	51
NCI-SEER	65	298	2	23	-	2	-	-	1	3	-	-	62	270	332
NSW	34	157	-	2	-	1	-	-	-	-	-	-	34	154	188
SCALE	64	299	-	6	-	-	-	-	-	2	-	-	64	291	355
UCSF2	94	10	1	0	-	-	-	-	2	-	-	-	91	10	101
YALE	28	149	-	3	-	-	-	-	-	-	-	-	28	146	174
**Subtotal**	335	1328	7	48	-	5	-	-	3	5	-	-	325	1270	1595
**Clinic or hospital-based or mixed case-control studies**				-	-	-	-	-	-	-	-	-	-	-	-
ENGELA	8	77	3	14	-	-	-	-	-	-	-	-	5	63	68
EpiLymph	71	250	12	35	-	-	-	1	-	3	-	-	59	211	270
Iowa-Mayo SPORE	113	-	1	0	-	-	-	-	-	-	-	-	112	-	112
Mayo Case-Control	78	357	2	12	-	-	-	1	1	1	-	-	75	343	418
MSKCC	67	9	18	5	-	-	-	-	2	-	-	-	47	4	51
**Subtotal**	337	693	36	66	-	-	-	2	3	4	-	-	298	621	919
**Grand total**	**890**	**2854**	**55**	**148**	**-**	**6**	**3**	**2**	**7**	**13**	**-**	**3536**	**825**	**6221**	**7046**

Supplementary Table 3. Characteristics and summary of the cases and controls included in the final analysis for MZL GWAS and replication

	No. of subjects		% Male		Mean age (SD)	
**Study**	**Cases**	**Controls**	**Cases**	**Controls**	**Cases**	**Controls**
**STAGE 1 GWAS**						
ATBC	1	240	100.0%	100.0%	61.00 (0.00)	68.35 (7.67)
BCCA	40	109	47.5%	56.0%	65.05 (10.75)	60.70 (12.66)
CPS-II	52	220	50.0%	49.5%	72.96 (7.33)	68.41 (6.28)
EPIC	8	265	37.5%	45.3%	59.00 (7.38)	62.65 (8.42)
ENGELA	5	63	100.0%	65.1%	58.40 (7.39)	55.25 (11.34)
EpiLymph	59	211	35.6%	54.0%	59.93 (14.24)	59.31 (12.96)
HPFS	5	85	100.0%	100.0%	64.20 (8.16)	70.56 (8.43)
Iowa-Mayo SPORE	112	0	47.3%	NA	63.51(10.35)	NA
Italian GxE	6	45	66.7%	62.2%	55.83 (14.68)	55.38 (11.68)
MAYO Case-control	75	343	41.3%	61.2%	62.88 (11.47)	60.90 (13.35)
MCCS	8	75	50.0%	52.0%	68.88 (8.25)	70.96 (7.85)
MSKCC	47	4	36.2%	0.0%	59.17 (11.78)	41.25 (9.36)
NCI-SEER	62	270	51.6%	54.1%	60.21 (13.11)	56.57 (11.94)
NHS	12	88	0.0%	0.0%	71.92 (3.35)	64.01 (6.91)
NSW	34	154	44.1%	60.4%	59.56 (11.71)	57.19 (11.13)
NYU-WHS	6	53	0.0%	0.0%	74.33(7.99)	76.17 (9.20)
PLCO	26	3076	53.8%	95.7%	71.31 (7.21)	69.54 (6.19)
SCALE	64	291	57.8%	57.7%	60.33(9.95)	60.01(12.12)
UCSF2	91	10	51.6%	60.0%	60.33 (14.73)	43.40 (14.83)
ELCCS	0	245	NA	50.6%	NA	53.19 (8.19)
WHI	84	228	0.0%	0.0%	72.90(7.16)	77.66(6.58)
YALE	28	146	0.0%	0.0%	63.57 (11.47)	61.68 (13.47)
Total	825	6221	40.5%	72.8%	63.94(12.18)	66.04(10.44)
**REPLICATION**						
MD Anderson	74	76	39.19%	39.47%	59.32 (10.08)	59.26 (9.87)
MSKCC	223	378	43.95%	17.99%	61.09 (13.55)	57.80 (11.69)
MAYO	115	383	47.83%	57.96%	60.41 (13.54)	62.13 (12.33)
NCI Replication	44	69	56.82%	39.13%	65.75 (10.75)	60.80 (14.79)
Total	456	906	45.39%	38.30%	61.08 (12.86)	59.98 (12.23)
**Grand Total**	1281	7127	42.23%	68.39%	62.92 (12.50)	65.27 (10.87)

Supplementary Table 4. Association with MZL for all SNPs taken forward for replication

SNP	Chr	Position[a]	Risk allele[b]	Other allele	RAF[c]	Genotyped/ Imputed[d]	Stage	No. of cases	No. of controls	OR	(95% CI)	*P*	$P_{heterogeneity}$	I^2
rs9461741	6	32370587	C	G	0.018	i (0.999)	Stage 1	824	6220	2.40	(1.74-3.31)	9.11E-08		
					0.030	g	Stage 2	453	877	3.06	(2.10-4.46)	5.24E-09		
							**Combined**	**1277**	**7097**	**2.66**	**(2.08-3.39)**	**3.95E-15**	**0.216**	**34.69**
rs2922994	6	31335901	G	A	0.113	i (1.00)	Stage 1	825	6221	1.74	(1.43-2.12)	2.89E-08		
					0.094	g	Stage 2	405	832	1.43	(1.08-1.90)	0.01		
							**Combined**	**1230**	**7053**	**1.64**	**(1.39-1.92)**	**2.43E-09**	**0.507**	**0**
rs7750641	6	31129310	T	C	0.113	g	Stage 1	825	6221	1.68	(1.38-2.04)	2.57E-07		
					0.095	g	Stage 2	451	900	1.38	(1.05-1.80)	0.02		
							**Combined**	**1276**	**7121**	**1.57**	**(1.34-1.84)**	**3.34E-08**	**0.186**	**40.52**
rs9268671	6	32414290	G	A	0.685	i (0.992)	Stage 1	825	6220	1.39	(1.23-1.56)	5.53E-08		
					0.312	g	Stage 2	445	894	0.96	(0.81-1.15)	0.66		
							**Combined**	**1270**	**7114**	**1.27**	**(1.15-1.41)**	**1.91E-06**	**0.001**	**85.15**
rs76788097	14	61729493	G	A	0.062	i (0.986)	Stage 1	824	6221	1.63	(1.32-2.01)	6.64E-06		
					0.064	g	Stage 2	447	897	1.33	(0.97-1.83)	0.08		
							**Combined**	**1271**	**7118**	**1.53**	**(1.28-1.82)**	**2.31E-06**	**0.244**	**29.10**
rs76588427	2	21135687	A	G	0.017	i (0.989)	Stage 1	825	6220	2.38	(1.65-3.44)	4.02E-06		
					0.022	g	Stage 2	448	901	1.53	(0.92-2.55)	0.10		
							**Combined**	**1273**	**7121**	**2.05**	**(1.52-2.76)**	**2.70E-06**	**0.209**	**36.06**
rs1202393	7	148915298	A	G	0.139	i (0.998)	Stage 1	825	6220	1.41	(1.21-1.64)	7.05E-06		
					0.157	g	Stage 2	450	896	1.08	(0.86-1.36)	0.51		
							**Combined**	**1275**	**7116**	**1.30**	**(1.15-1.48)**	**3.60E-05**	**0.002**	**84.22**
rs55825400	8	76068956	A	G	0.025	i (0.891)	Stage 1	824	6220	2.41	(1.67-3.48)	2.43E-06		
					0.026	g	Stage 2	449	904	1.21	(0.72-2.04)	0.47		
							**Combined**	**1273**	**7124**	**1.92**	**(1.42-2.59)**	**1.94E-05**	**0.043**	**68.33**
rs6768604	3	185704345	A	G	0.279	g	Stage 1	825	6221	1.35	(1.20-1.54)	8.59E-07		
					0.270	g	Stage 2	452	895	0.93	(0.77-1.12)	0.45		
							**Combined**	**1277**	**7116**	**1.21**	**(1.10-1.35)**	**0.0002**	**0.004**	**82.03**
rs59059443	5	29727289	T	C	0.037	i (0.813)	Stage 1	824	6220	2.13	(1.55-2.93)	2.72E-06		
					0.055	g	Stage 2	451	895	0.89	(0.60-1.31)	0.54		
							**Combined**	**1275**	**7115**	**1.50**	**(1.17-1.91)**	**0.001**	**0.001**	**84.64**

[a]Position according to human reference NCBI37/hg19; [b]Allele associated with an increased risk of MZL; [c]Risk allele frequency in controls;[d]g=genotyped, i=imputed (info) p-values and odds-ratios were generated by using logistic regression. Heterogeneity in the effect estimates was assessed using Cochran's Q statistic and estimating the I^2 statistic.

Supplementary Table 5. Risk associated with MZL for SNPs in the HLA region previously reported to be associated with other lymphoma subtypes

SNP	Position	Effect allele	Other allele	Genotyped /Imputed	EAF	No. of cases	No. of controls	OR	95% CI	*P*	NHL subtype association	PubMed ID	Reference
rs6457327	31074030	C	A	g	0.617	825	6221	1.05	(0.94-1.18)	0.37	FL	19620980	Skibola CF *et al.*
rs2248462	31446796	A	G	g	0.209	825	6221	0.88	(0.77-1.01)	0.07	HD	22286212	Urayama KY *et al.*
rs3132453	31604044	G	T	g	0.933	825	6221	0.98	(0.78-1.23)	0.86	B-cell lymphoma	23047821	Nieters A *et al.*
rs204999	32109979	G	A	g	0.261	825	6221	0.97	(0.84-1.10)	0.67	NS-HD	22086417	Cozen W *et al.*
rs926070	32257566	A	G	g	0.681	825	6221	0.90	(0.80-1.01)	0.08	CLL	24292274	Speedy HE *et al.*
rs9268528	32383108	G	A	g	0.379	825	6221	0.88	(0.79-0.98)	0.02	NS-HD	22086417	Cozen W *et al.*
rs9268542	32384721	G	A	g	0.383	825	6221	0.87	(0.78-0.97)	0.02	NS-HD	22086417	Cozen W *et al.*
rs6903608	32428285	T	C	g	0.680	825	6221	0.96	(0.85-1.07)	0.44	HD	21037568	Enciso-Mora V *et al.*
rs9268853	32429643	C	T	g	0.306	825	6221	0.92	(0.82-1.04)	0.17	Lymphoma	23349640	Vijai J *et al.*
rs2395185	32433167	T	G	g	0.306	825	6221	0.92	(0.82-1.04)	0.17	HD	22286212	Urayama KY *et al.*
rs2858870	32572251	C	T	i (0.937)	0.108	824	6220	0.84	(0.71-1.01)	0.06	NS-HD	22086417	Cozen W *et al.*
rs674313	32578082	T	C	i (0.997)	0.260	825	6220	1.14	(1.01-1.30)	0.04	CLL	21131588	Slager SL *et al.*
rs4530903	32581889	T	C	g	0.114	825	6221	1.03	(0.87-1.21)	0.77	Lymphoma	23349640	Vijai J *et al.*
rs9272535	32606756	A	G	i (0.952)	0.263	824	6220	1.18	(1.04-1.33)	0.01	CLL	21131588	Slager SL *et al.*
rs9273363	32626272	A	C	g	0.270	825	6221	1.05	(0.93-1.19)	0.44	CLL	23770605	Berndt SI *et al.*
rs2647012	32664458	C	T	i (1.0)	0.601	825	6221	0.89	(0.79-0.99)	0.04	FL	21533074	Smedby KE *et al.*
rs10484561	32665420	G	T	i (1.0)	0.121	825	6221	0.99	(0.84-1.17)	0.92	FL	20639881	Conde L *et al.*
rs2647045	32668100	A	G	i (0.996)	0.236	825	6221	0.94	(0.83-1.06)	0.30	Lymphoma	23349640	Vijai J *et al.*
rs2621416	32741868	C	T	g	0.276	825	6221	0.93	(0.83-1.05)	0.27	Lymphoma	23349640	Vijai J *et al.*
rs241447	32796751	C	T	g	0.269	825	6221	0.94	(0.83-1.06)	0.28	FL	2291133	Cerhan J *et al.*

Abbreviations: HD: Hodgkin's lymphoma; CLL: Chronic Lymphocytic leukemia; NS-HD: Nodular sclerosing Hodgkin's lymphoma; FL: Follicular Lymphoma; EAF: Effect Allele Frequency; OR: Odds-ratio; P: p-value

Supplementary Table 6. Associations with MZL for the HLA allele and amino acid position imputed from the GWAS data

Marker	Risk allele	Other allele	MAF	INFO	OR	95% CI	*P*
*HLA-B*0801*	Pr	Ab	0.115	1.02	1.67	(1.38-2.01)	7.79E-08
HLA-B Asp at position 9	Pr	Ab	0.115	1.02	1.67	(1.38-2.01)	7.94E-08
*HLA-B*08*	Pr	Ab	0.115	1.02	1.67	(1.38-2.01)	7.94E-08
*HLA-DRB1*0102*	Pr	Ab	0.017	1.06	2.24	(1.64-3.07)	5.08E-07
HLA-DRβ*1* Ala at position 85	Ala	Val	0.035	1.00	1.84	(1.44-2.34)	7.80E-07

Alleles: Pr=present, Ab=absent, Ala=Alanine, Val=Valine, MAF=Minor Allele Frequency, INFO= marker imputation quality information, OR=odds-ratio and P=p-value
Logistic regression analysis using the log-additive model and age of onset, gender and significant eigenvalues used as covariates.

Supplementary Table 7. Results for the two novel loci stratified by MZL subtype (MALT vs. non-MALT)

				MALT				Non-MALT				
SNP	Risk allele[a]	Other allele	RAF[b]	No. of cases/ No. of controls	OR	95% CI	*P*	No. of cases/ No. of controls	OR	95% CI	*P*	$P_{heterogeneity}$
rs2922994	G	A	0.11	358/6221	1.92	(1.50-2.47)	2.43E-07	467/6221	1.58	(1.23-2.03)	0.0004	0.6
rs9461741	C	G	0.02	358/6220	2.95	(1.89-4.62)	2.19E-06	466/6220	2.44	(1.57-3.79)	7.27E-05	0.05

[a]Allele associated with an increased risk of MZL; [b]Risk allele frequency in controls. p-values and odds-ratios were generated by using logistic regression . Heterogeneity in the effect estimates was assessed using Cochran's Q statistic and estimating the I^2 statistic.

Supplementary Table 8. Association results for *H.pylori*-associated SNPs with MZL and MALT

Group	SNP	Chr	Position	Other Allele	Effect Allele	f-Case	f-Ctrl	OR	(95% CI)	*P*
MZL	rs4833103	4	38815502	A	C	0.526	0.465	1.15	(1.03-1.28)	0.01
MZL	rs11568818	11	102401661	T	C	0.479	0.449	1.14	(1.03-1.27)	0.01
MZL	rs2066844	16	50745926	C	T	0.033	0.045	0.74	(0.58-0.96)	0.02
MALT	rs4833103	4	38815502	A	C	0.5098	0.4654	1.04	(0.89-1.22)	0.59
MALT	rs11568818	11	102401661	T	C	0.4679	0.4495	1.09	(0.94-1.28)	0.26
MALT	rs2066844	16	50745926	C	T	0.0405	0.0454	0.90	(0.62-1.30)	0.58

Chr: chromosome; Position: hg19 coordinate start, f-Case: allele frequency in cases, f-Ctrl: allele frequency in controls, OR=odds-ratio and P=p-value
Logistic regression analysis using the log-additive model and age of onset, gender and significant eigenvalues used as covariates.

Supplementary Table 9. eQTL associations for the MZL SNPs from a childhood asthma study (data from Dixon et al.)*

MZL SNP	Gene transcript	Effect allele	Other allele	Beta for MZL SNP[a]	*P* for MZL SNP[a]	*P* for MZL SNP conditioned on peak SNP[b]	Peak SNP for transcript[c]	Beta for Peak SNP[d]	*P* for Peak SNP[d]	*P* for peak SNP conditioned on MZL SNP[e]
rs2922994	*HLA-C*	A	G	-2.564	7.06E-11	9.06E-06	rs140242258	0.836	7.36E-25	1.31E-22
rs7750641	*HLA-C*	C	T	-0.544	1.02E-12	3.98E-08	rs140242258	0.836	7.36E-25	1.53E-22
rs2922994	*HLA-B*	A	G	-1.225	1.59E-05	0.013	rs140242258	0.461	3.51E-15	5.31E-13
rs7750641	1557242_at	C	T	-0.443	3.12E-06	3.42E-05	rs80296299	0.988	5.23E-12	1.97E-10

[*]See Methods for details
[a]Beta and p-value for the association between the MZL SNP and gene transcript.
[b]p-value for the association between the MZL SNP and gene transcript after adjustment for the peak SNP
[c]Peak SNP is the most significant SNP associated with the gene transcript
[d]Beta and p-value for the association between the peak SNP and the gene transcript
[e]P-value for the association between the peak SNP and the gene transcript after adjustment for the MZL SNP

Supplementary Table 10. eQTL associations with the correlated SNP for the HLA SNP rs2922994. Only those with FDR p-value≤0.05 are shown.

SNP	CHR:POS	r^2	Probe	*P*	FDR
rs2524069	6:31244789	0.86	RNF5:NM_006913.RNF5	3.16E-06	0.006
rs2844613	6:31243846	0.86	RNF5:NM_006913.RNF5	2.55E-06	0.006
rs2524067	6:31245821	0.86	RNF5:NM_006913.RNF5	3.16E-06	0.006
rs3099844	6:31448976	0.92	RNF5:NM_006913.RNF5	5.55E-05	0.024
rs2596565	6:31353329	1	RNF5:NM_006913.RNF5	5.55E-05	0.024
rs3132510	6:31172151	1	RNF5:NM_006913.RNF5	5.55E-05	0.024
rs3131643	6:31442782	0.84	RNF5:NM_006913.RNF5	5.55E-05	0.024
rs3134792	6:31312326	1	RNF5:NM_006913.RNF5	5.55E-05	0.024
rs2524078	6:31242649	0.81	RNF5:NM_006913.RNF5	1.95E-05	0.024
rs3094005	6:31465047	0.92	RNF5:NM_006913.RNF5	5.55E-05	0.024
rs2734583	6:31505480	0.92	RNF5:NM_006913.RNF5	5.55E-05	0.024
rs3131618	6:31434621	0.92	RNF5:NM_006913.RNF5	5.55E-05	0.024
rs9266669	6:31348077	0.87	RNF5:NM_006913.RNF5	3.94E-05	0.024
rs2844577	6:31334422	1	LY6G6E:NR_003673.LY6G6E	7.22E-05	0.029
rs1634726	6:30985828	0.81	LY6G6E:NR_003673.LY6G6E	9.99E-05	0.037
rs2524069	6:31244789	0.86	HLA-E:NM_005516.HLA-E	1.83E-04	0.045
rs2524069	6:31244789	0.86	FLOT1:NM_005803.FLOT1	3.62E-04	0.045
rs2844613	6:31243846	0.86	HLA-E:NM_005516.HLA-E	2.41E-04	0.045
rs2844613	6:31243846	0.86	FLOT1:NM_005803.FLOT1	2.41E-04	0.045
rs4143332	6:31348365	1	FLOT1:NM_005803.FLOT1	3.17E-04	0.045
rs4143332	6:31348365	1	RNF5:NM_006913.RNF5	2.76E-04	0.045
rs3099844	6:31448976	0.92	FLOT1:NM_005803.FLOT1	3.72E-04	0.045
rs2524067	6:31245821	0.86	HLA-E:NM_005516.HLA-E	1.83E-04	0.045
rs2524067	6:31245821	0.86	FLOT1:NM_005803.FLOT1	3.62E-04	0.045
rs3132473	6:31408329	0.9	LY6G6E:NR_003673.LY6G6E	1.71E-04	0.045
rs2596565	6:31353329	1	FLOT1:NM_005803.FLOT1	3.72E-04	0.045
rs3132510	6:31172151	1	FLOT1:NM_005803.FLOT1	3.72E-04	0.045
rs3131643	6:31442782	0.84	FLOT1:NM_005803.FLOT1	3.72E-04	0.045
rs3130923	6:31462135	0.84	FLOT1:NM_005803.FLOT1	2.47E-04	0.045
rs3132472	6:31386131	0.92	FLOT1:NM_005803.FLOT1	3.17E-04	0.045
rs3132472	6:31386131	0.92	RNF5:NM_006913.RNF5	2.76E-04	0.045
rs2844559	6:31340075	1	RNF5:NM_006913.RNF5	2.15E-04	0.045
rs3134792	6:31312326	1	FLOT1:NM_005803.FLOT1	3.72E-04	0.045
rs2524078	6:31242649	0.81	FLOT1:NM_005803.FLOT1	3.55E-04	0.045
rs2922994	6:31335901	NA	FLOT1:NM_005803.FLOT1	3.17E-04	0.045
rs2922994	6:31335901	NA	RNF5:NM_006913.RNF5	2.76E-04	0.045
rs3130614	6:31476458	0.92	FLOT1:NM_005803.FLOT1	3.17E-04	0.045
rs3130614	6:31476458	0.92	RNF5:NM_006913.RNF5	2.76E-04	0.045
rs3094005	6:31465047	0.92	FLOT1:NM_005803.FLOT1	3.72E-04	0.045
rs2734583	6:31505480	0.92	FLOT1:NM_005803.FLOT1	3.72E-04	0.045
rs3131618	6:31434621	0.92	FLOT1:NM_005803.FLOT1	3.72E-04	0.045
rs9266669	6:31348077	0.87	FLOT1:NM_005803.FLOT1	2.65E-04	0.045
rs2596430	6:31335431	1	FLOT1:NM_005803.FLOT1	3.17E-04	0.045
rs2596430	6:31335431	1	RNF5:NM_006913.RNF5	2.76E-04	0.045
rs2156874	6:31335976	1	FLOT1:NM_005803.FLOT1	3.17E-04	0.045
rs2156874	6:31335976	1	RNF5:NM_006913.RNF5	2.76E-04	0.045
rs3130612	6:31482097	0.92	RNF5:NM_006913.RNF5	3.86E-04	0.046

rs2524078	6:31242649	0.81	DPCR1:NM_080870.DPCR1	4.11E-04	0.048

Supplementary Table 11. Functional motif annotations from HaploReg analysis of newly-discovered MZL risk loci and their correlated (r^2 > 0.80) SNPs

chr	pos (hg19)	LD (r^2)	variant	Ref	Alt	EUR freq	SiPhy cons	Promoter histone marks	Enhancer histone marks	DNAse	Proteins bound	Motifs changed	GENCODE genes	dbSNP func annot	SNP in LD
6	31326074	0.87	rs2853999	A	T	0.09		HepG2	GM12878, NHLF	CD34+_Mobilized	POL2	STAT	1.1kb 5' of HLA-B		rs2156874
6	31326703	0.91	rs2523593	T	C	0.09			NHLF, GM12878	Hepatocytes		E2A,Myc,Pax-4	1.7kb 5' of HLA-B		rs2156874
6	31327890	0.95	rs2523584	A	G	0.09						6 altered motifs	2.9kb 5' of HLA-B		rs2156874
6	31328988	0.95	rs2523573	C	G	0.09						4 altered motifs	4kb 5' of HLA-B		rs2156874
6	31329374	0.95	rs2596545	A	C	0.09						5 altered motifs	4.4kb 5' of HLA-B		rs2156874
6	31329642	0.95	rs2596547	C	T	0.09						AP-1,Foxa,Foxj1	4.7kb 5' of HLA-B		rs2156874
6	31329691	0.95	rs2523571	T	A	0.09						5 altered motifs	4.7kb 5' of HLA-B		rs2156874
6	31332920	1	rs2523546	G	A	0.09						Pax-5,Pax-8,SRF	5kb 5' of U6		rs2156874
6	31334422	1	rs2844577	T	C	0.09		6 cell types	H1, HMEC	AoSMC		Pou6f1	3.5kb 5' of U6		rs2156874
6	31335431	1	rs2596430	T	C	0.09						HDAC2,HNF4	2.5kb 5' of U6		rs2156874
6	31335901	1	rs2922994	A	G	0.09						4 altered motifs	2kb 5' of U6		rs2156874
6	31335976	1	rs2156874	G	C	0.09						CEBPB,Cdc5,Mef2	1.9kb 5' of U6		rs2156874
6	31338844	0.86	rs2853986	T	C	0.09							826bp 3' of U6		rs2156874
6	31339996	0.94	rs2523524	G	A	0.09						ELF1,GR,p300	2kb 3' of U6		rs2156874
6	31340075	0.94	rs2844559	C	T	0.09							2kb 3' of AL671883.1		rs2156874
6	31344390	0.83	rs2905719	A	G	0.09				Osteobl		COMP1	2.2kb 5' of AL671883.1		rs2156874
6	31346653	0.85	rs2442735	A	G	0.09		HepG2		5 cell types		HIF1,Pax-5,Zfp161	4.5kb 5' of AL671883.1		rs2156874
6	31346849	0.85	rs11433356	CG	C	0.09			HepG2			GR,Hmx,SREBP	4.7kb 5' of AL671883.1		rs2156874
6	31348365	0.85	rs4143332	G	A	0.09						TLX1::NFIC	6.2kb 5' of AL671883.1		rs2156874
6	31348519	0.85	rs4143333	A	G	0.09						Nkx2	6.3kb 5' of AL671883.1		rs2156874
6	31353171	0.8	rs2844531	A	G	0.09						STAT	11kb 5' of AL671883.1		rs2156874
6	31353329	0.8	rs2596565	G	A	0.09				pHTE		SP1	11kb 5' of AL671883.1		rs2156874
6	31353435	0.8	rs2844530	C	G	0.09				pHTE		CCNT2,EWSR1-FLI1,UF1H3BETA	11kb 5' of AL671883.1		rs2156874
6	31348822	0.86	rs201523396	AAT	A	0.08						10 altered motifs	6.6kb 5' of AL671883.1		rs2442735
6	31348830	0.84	rs200032555	TA	T	0.08						31 altered motifs	6.6kb 5' of AL671883.1		rs2442735
6	31462135	0.87	rs3130923	G	A	0.08		9 cell types		25 cell types	5 bound proteins	7 altered motifs	522bp 5' of MICB		rs2442735
6	31465047	0.87	rs3094005	G	T	0.08		GM12878, HepG2, H1	6 cell types	HMEC		5 altered motifs	MICB		rs2442735
6	31469774	0.87	rs3095233	A	G	0.08			GM12878			Bbx,Sox	MICB	intronic	rs2442735
6	31473746	0.9	rs3130616	G	A	0.08						21 altered motifs	MICB	intronic	rs2442735
6	31473957	0.83	rs3134900	C	G	0.09						ATF3,NRSF	MICB	missense	rs2442735
6	31476458	0.88	rs3130614	T	A	0.08						Ets,Mef2	MICB	intronic	rs2442735
6	31482097	0.9	rs3130612	T	G	0.08						6 altered motifs	3.2kb 3' of MICB		rs2442735
6	31483481	0.92	rs9267445	G	C	0.08						HNF4,Rad21	4.6kb 3' of MICB		rs2442735
6	31505480	1	rs2734583	A	G	0.08				Fibrobl,WERI-Rb-1			ATP6V1G2-DDX39B	intronic	rs2442735
6	31514247	1	rs9267488	A	G	0.08		4 cell types	5 cell types		KAP1	4 altered motifs	ATP6V1G2	intronic	rs2442735
6	31546850	0.93	rs1800628	G	A	0.08			NHEK, GM12878, HMEC	GM12864	POL24H8,POL2	6 altered motifs	736bp 3' of TNF		rs2442735

6	31558702	0.9	rs3130063	C	T	0.08						Rad21,SMC3	NCR3	intronic	rs2442735
6	31566168	0.93	rs3130631	C	G	0.08		HepG2	GM12878	HepG2	5 bound proteins	HIF1,HNF4	5.4kb 5' of NCR3		rs2442735
18	53721590	0.92	rs1970748	A	G	0.51						4 altered motifs	AC006305.1		rs2852986
18	53733950	0.99	rs2109685	G	A	0.51				NHDF-Ad			AC006305.1		rs2852986
18	53734603	0.99	rs2535726	A	G	0.51			HSMM	19 cell types	CTCF,P300	GR,STAT	AC006305.1		rs2852986
18	53735471	0.99	rs2535727	G	A	0.51			4 cell types	4 cell types		5 altered motifs	AC006305.1		rs2852986
18	53735635	0.99	rs2535728	G	T	0.51			4 cell types	14 cell types		NF-I,Nanog,Pou2f2	AC006305.1		rs2852986
18	53736180	0.9	rs2535729	G	A	0.49			4 cell types	5 cell types		9 altered motifs	AC006305.1		rs2852986
18	53740116	1	rs2852986	G	A	0.51						Sox	AC006305.1		rs2852986
18	53740183	0.99	rs1073719	T	C	0.51						p300	AC006305.1		rs2852986
6	31436738	0.92	rs76650611	A	T	0.08			GM12878				HCP5		rs3099844
6	31448564	1	rs3099843	G	T	0.09						4 altered motifs	3.3kb 3' of HCP5		rs3099845
6	31448976	1	rs3099844	C	A	0.09			HepG2			6 altered motifs	3.7kb 3' of HCP5		rs3099846
6	31449552	0.86	rs3130908	T	C	0.08			HepG2			11 altered motifs	4.3kb 3' of HCP5		rs3099847
6	31449710	0.82	rs3130909	T	C	0.09			HepG2			6 altered motifs	4.4kb 3' of HCP5		rs3099848
6	31450637	1	rs3131642	G	A	0.09							5.4kb 3' of HCP5		rs3099849
6	31451370	1	rs3132470	A	G	0.09				HIPEpiC,SKMC	CEBPB	CEBPA,CEBPB	6.1kb 3' of HCP5		rs3099850
6	31451836	1	rs3094011	T	C	0.09						Pou3f3	6.6kb 3' of HCP5		rs3099851
6	31451848	1	rs3094010	C	T	0.09						Ets	6.6kb 3' of HCP5		rs3099852
6	31377978	1	rs2523495	C	T	0.09						Pax-5,SETDB1	MICA	intronic	rs3132472
6	31386131	1	rs3132472	G	A	0.09				HFF		4 altered motifs	HCP5		rs3132472
6	31408329	1	rs3132473	T	A	0.09						CAC-binding-protein,Foxj2	HCP5		rs3132473
6	31410521	0.98	rs3093958	A	G	0.08		HepG2		40 cell types	4 bound proteins		XXbac-BPG181B23.4		rs3132473
6	31414051	0.98	rs141516569	CTTTTT	C	0.08						14 altered motifs	XXbac-BPG181B23.4		rs3132473
6	31414241	0.98	rs9267092	G	T	0.09						Mef2	XXbac-BPG181B23.4		rs3132473
6	31427395	0.85	rs9267123	G	C	0.1							HCP5		rs3132473
6	31428920	0.93	rs3130477	T	C	0.09				FibroP,NHDF-Ad,WI-38		4 altered motifs	HCP5		rs3132473
6	31430010	0.93	rs3132089	G	A	0.09			GM12878, NHEK, NHLF			11 altered motifs	HCP5		rs3132473
6	31430065	0.93	rs3099839	C	T	0.09		H1	5 cell types			Pou2f2,Smad3	HCP5		rs3132473
6	31430694	0.93	rs3094605	G	C	0.09		H1, HepG2	4 cell types		5 bound proteins	4 altered motifs	HCP5		rs3132473
6	31430752	0.93	rs3132090	G	A	0.09		H1, HepG2	4 cell types	NHEK,Caco-2	11 bound proteins	8 altered motifs	HCP5		rs3132473
6	31431813	0.93	rs3130907	A	G	0.09		GM12878, NHEK	HepG2		POL2	RXRA	HCP5	3'-UTR	rs3132473

6	31433693	0.93	rs3128986	T	C	0.09	GM12878		POL2	9 altered motifs	HCP5	rs3132473
6	31434331	0.93	rs3131619	A	T	0.09			POL2	Pax-5	HCP5	rs3132473
6	31434366	0.93	rs3094013	G	A	0.09			POL2	AIRE	HCP5	rs3132473
6	31434520	0.86	rs3094012	G	C	0.09		Osteobl	POL2,POL24H8	4 altered motifs	HCP5	rs3132473
6	31434621	0.92	rs3131618	A	G	0.08			POL2	CDP	HCP5	rs3132473
6	31300197	0.8	rs79158921	G	A	0.09				Nkx2	21kb 3' of HLA-B	rs3134792
6	31306420	0.83	rs1625792	G	A	0.09				Mef2	15kb 3' of HLA-B	rs3134792
6	31306639	0.8	rs1634753	C	T	0.1				BDP1,Dobox4	15kb 3' of HLA-B	rs3134792
6	31308476	0.86	rs2854019	T	A	0.09			KAP1	AP-1,Sox	13kb 3' of HLA-B	rs3134792
6	31308562	0.82	rs2854018	C	T	0.09				5 altered motifs	13kb 3' of HLA-B	rs3134792
6	31308717	0.86	rs402175	G	A	0.09				Gfi1,Pax-4	13kb 3' of HLA-B	rs3134792
6	31308988	0.85	rs1634776	C	T	0.09				BDP1,SETDB1	13kb 3' of HLA-B	rs3134792
6	31309785	0.88	rs3132474	G	A	0.1				Sox	12kb 3' of HLA-B	rs3134792
6	31310372	0.84	rs9265811	C	T	0.1				DMRT5,Irf	11kb 3' of HLA-B	rs3134792
6	31311318	0.84	rs9265827	A	G	0.11				4 altered motifs	10kb 3' of HLA-B	rs3134792
6	31311449	0.92	rs9265831	T	A	0.09				Pou2f2,Pou3f3,Smad	10kb 3' of HLA-B	rs3134792
6	31311912	0.8	rs2394976	G	T	0.11				5 altered motifs	9.7kb 3' of HLA-B	rs3134792
6	31311950	1	rs2256747	T	C	0.09				GR,Hoxa7,Rad21	9.7kb 3' of HLA-B	rs3134792
6	31312020	1	rs2256750	G	A	0.09				7 altered motifs	9.6kb 3' of HLA-B	rs3134792
6	31312259	1	rs2844589	G	A	0.09					9.4kb 3' of HLA-B	rs3134792
6	31312326	1	rs3134792	T	G	0.09				HEN1,PEBP	9.3kb 3' of HLA-B	rs3134792
6	31312492	1	rs2854010	A	G	0.09				SRF	9.2kb 3' of HLA-B	rs3134792
6	31312607	1	rs9265857	G	C	0.09				5 altered motifs	9kb 3' of HLA-B	rs3134792
6	31312656	0.98	rs2394978	C	T	0.09				AP-1,HNF4,ZEB1	9kb 3' of HLA-B	rs3134792
6	31312729	0.98	rs2394979	G	A	0.09				E2F,Irx,SRF	8.9kb 3' of HLA-B	rs3134792
6	31312941	0.98	rs2394980	C	T	0.09		6 cell types	CTCF	Gfi1,Gfi1b,TATA	8.7kb 3' of HLA-B	rs3134792
6	31313029	0.86	rs2394981	G	T	0.09		6 cell types	CTCF	4 altered motifs	8.6kb 3' of HLA-B	rs3134792
6	31313221	0.94	rs9265886	A	C	0.09		HPAEC		CTCF	8.4kb 3' of HLA-B	rs3134792
6	31313367	0.94	rs7450305	T	C	0.09		AoAF,HPAF		GLI,Hoxa7,Pax-1	8.3kb 3' of HLA-B	rs3134792
6	31313677	1	rs9265908	C	T	0.09				HDAC2,Pax-5	8kb 3' of HLA-B	rs3134792
6	31313722	0.97	rs9265910	C	T	0.09				4 altered motifs	7.9kb 3' of HLA-B	rs3134792
6	31314274	1	rs7749555	A	G	0.09				CEBPB	7.4kb 3' of HLA-B	rs3134792
6	31314652	1	rs2507998	A	G	0.09				7 altered motifs	7kb 3' of HLA-B	rs3134792
6	31314938	0.97	rs9265937	C	T	0.09				Brachyury,Irf,SREBP	6.7kb 3' of HLA-B	rs3134792
6	31315005	0.95	rs9265938	C	T	0.09					6.6kb 3' of HLA-B	rs3134792
6	31315027	0.95	rs9265939	A	G	0.09				HNF1,YY1	6.6kb 3' of HLA-B	rs3134792
6	31315058	0.95	rs9265942	C	G	0.09				Sox,TEF	6.6kb 3' of HLA-B	rs3134792
6	31315138	0.95	rs9265946	G	A	0.09				NRSF	6.5kb 3' of HLA-B	rs3134792
6	31315193	0.95	rs9265948	C	T	0.09				4 altered motifs	6.5kb 3' of HLA-B	rs3134792
6	31315229	0.92	rs9265949	C	T	0.09				Foxp1,Pou2f2	6.4kb 3' of HLA-B	rs3134792
6	31315339	0.97	rs9265954	C	T	0.09				Pdx1	6.3kb 3' of HLA-B	rs3134792
6	31315618	0.94	rs9265964	A	G	0.09				5 altered motifs	6kb 3' of HLA-B	rs3134792
6	31315629	0.94	rs9265965	T	C	0.09		H7-hESC		4 altered motifs	6kb 3' of HLA-B	rs3134792

6	31315649	0.9	rs9265967	G	T	0.08			H7-hESC		4 altered motifs	6kb 3' of HLA-B		rs3134792
6	31315663	0.94	rs9265968	A	T	0.09			H7-hESC		GCNF,Pax-5,RXRA	6kb 3' of HLA-B		rs3134792
6	31315792	0.97	rs9265971	T	C	0.09					CDP	5.9kb 3' of HLA-B		rs3134792
6	31315805	0.97	rs9265972	G	C	0.09					CDP	5.8kb 3' of HLA-B		rs3134792
6	31315861	0.97	rs9265973	G	A	0.09					HNF4	5.8kb 3' of HLA-B		rs3134792
6	31315879	0.97	rs9265974	T	G	0.09					Pax-8,STAT	5.8kb 3' of HLA-B		rs3134792
6	31315933	0.97	rs9265976	A	G	0.09					8 altered motifs	5.7kb 3' of HLA-B		rs3134792
6	31316044	0.97	rs9265979	C	T	0.09					5 altered motifs	5.6kb 3' of HLA-B		rs3134792
6	31316080	0.92	rs9265982	C	T	0.09					BAF155,Osf2,PEBP	5.6kb 3' of HLA-B		rs3134792
6	31316234	0.97	rs9265985	A	C	0.09			PanIsletD	FOXA1	7 altered motifs	5.4kb 3' of HLA-B		rs3134792
6	31316448	0.97	rs9265990	T	A	0.09					Irf,STAT	5.2kb 3' of HLA-B		rs3134792
6	31316520	0.97	rs9265993	G	A	0.09					AIRE,Foxp3	5.1kb 3' of HLA-B		rs3134792
6	31316526	0.94	rs9265994	T	C	0.09					AIRE,TCF12	5.1kb 3' of HLA-B		rs3134792
6	31316613	0.97	rs9265998	G	A	0.09			GM19240,CD20+,GM12865		4 altered motifs	5kb 3' of HLA-B		rs3134792
6	31316695	0.94	rs9266001	C	A	0.09			GM19240,Adult_CD4_Th0,GM12865		Hmbox1,Pax-6	5kb 3' of HLA-B		rs3134792
6	31316911	0.94	rs2923008	G	T	0.09					4 altered motifs	4.7kb 3' of HLA-B		rs3134792
6	31317063	0.97	rs3016017	A	G	0.09					EBF	4.6kb 3' of HLA-B		rs3134792
6	31317065	0.97	rs2923007	T	C	0.09					CEBPB,Nanog,STAT	4.6kb 3' of HLA-B		rs3134792
6	31318308	0.95	rs2596510	G	T	0.09			WERI-Rb-1	POL24H8	Pax-2	3.3kb 3' of HLA-B		rs3134792
6	31318432	0.95	rs2596508	T	C	0.09		GM12878	GM19240,Melano,GM12865	POL2,POL24H8	Pax-5,Smad3,Smad	3.2kb 3' of HLA-B		rs3134792
6	31321267	0.84	rs2596500	A	C	0.09	GM12878		CD20+	POL24H8,POL2	Cdx,PLZF,RFX5	381bp 3' of HLA-B		rs3134792
6	31037872	0.8	rs200744629	TTTTC	T	0.08					7 altered motifs	10kb 3' of HCG22		rs7750641
6	31044463	0.81	rs3094671	T	C	0.09			Osteobl,SKMC		CEBPB,Hoxa5,Hoxa7	6.3kb 5' of U6		rs7750641
6	31051675	0.81	rs3095311	A	G	0.09		4 cell types	57 cell types	4 bound proteins	NF-I,Nanog	788bp 3' of U6		rs7750641
6	31058340	0.8	rs3130544	C	A	0.08					CHOP::CEBPalpha,ERalpha-a,RORalpha1	7.5kb 3' of U6		rs7750641
6	31079644	0.86	rs2233980	G	A	0.08		HSMM			Roaz	C6orf15	synonymous	rs7750641
6	31081434	0.9	rs3094222	A	G	0.09		4 cell types			Maf	1.1kb 5' of PSORS1C1		rs7750641
6	31085356	0.9	rs3130985	C	T	0.09	NHEK	4 cell types	Th1,PanIsletD,PanIslets	GATA3	6 altered motifs	CDSN	intronic	rs7750641
6	31094703	0.9	rs3130557	C	T	0.09		4 cell types	6 cell types		ERalpha-a,Gfi1,PU.1	PSORS1C1	intronic	rs7750641
6	31098734	0.9	rs3132541	A	C	0.09						PSORS1C1	intronic	rs7750641
6	31100974	0.9	rs3130562	T	C	0.09					13 altered motifs	PSORS1C1	intronic	rs7750641
6	31102618	0.85	rs3130566	C	G	0.1					Mef2	PSORS1C1	intronic	rs7750641
6	31102790	0.85	rs3132566	G	T	0.1					5 altered motifs	PSORS1C1	intronic	rs7750641

6	31103195	0.85	rs3094669	C	G	0.1						4 altered motifs	PSORS1C1	intronic	rs7750641
6	31105147	0.85	rs3131010	C	T	0.1			GM12878, HepG2			BDP1,GR,Pax-8	PSORS1C1	intronic	rs7750641
6	31129310	1	rs7750641	C	T	0.09			GM12878	HMEC,Osteobl	POL2	PTF1-beta,Rad21,ZBTB7A	TCF19	missense	rs7750641
6	31175946	0.8	rs1619179	A	C	0.09			HepG2			4 altered motifs	4.2kb 3' of HCG27		rs7750641
6	32353864	1	rs9268471	G	A							13 altered motifs	4.4kb 5' of HCG23		rs9268471
6	32339784	0.81	rs9501618	A	C	0.01			Huvec			CEBPB,PLZF,p300	99bp 5' of C6orf10		rs9461741
6	32342119	0.91	rs73729220	A	G	0.01					FOSL2, FOXA1		2.4kb 5' of C6orf10		rs9461741
6	32370587	1	rs9461741	G	C	0.01						7 altered motifs	BTNL2	intronic	rs9461741

Supplementary Table 12. Functional motif annotations from RegulomeDB analysis of newly-discovered MZL risk loci and their correlated (r^2 > 0.80) SNPs

#chr	coordinate	rsid	score	Transcription Factor Binding site, Factors used for ChIP-seq
chr6	31430751	rs3132090	2a	SMARCC1, RFX5, GTF2F1, IRF3, TFAP2C, TFAP2A, NFKB1, CEBPB, BRCA1, USF2, SP1
chr6	31462134	rs3130923	2b	SPI1, HEY1, TFAP2C, NANOG, E2F1
chr6	31430693	rs3094605	2b	SMARCC1, RFX5, TFAP2C, TFAP2A, NFKB1
chr6	31051674	rs3095311	2b	JUND, TRIM28, CEBPB, GTF3C2
chr6	31085355	rs3130985	2b	GATA3
chr6	31129309	rs7750641	2b	POLR2A
chr6	31566167	rs3130631	3a	HNF4A, HNF4G, USF1, RXRA, SP1
chr6	31433692	rs3128986	3a	POLR2A
chr6	31318431	rs2596508	3a	POLR2A
chr6	31546849	rs1800628	4	POLR2A
chr6	31451369	rs3132470	4	CEBPB
chr6	31410520	rs3093958	4	YY1, RAD21, CTCF, SMC3
chr6	31312940	rs2394980	4	CTCF
chr6	31313028	rs2394981	4	CTCF
chr6	31318307	rs2596510	4	POLR2A
chr6	31326073	rs2853999	5	POLR2A
chr6	31346652	rs2442735	5	
chr6	31465046	rs3094005	5	
chr6	31505479	rs2734583	5	
chr6	31514246	rs9267488	5	ZNF263, TRIM28
chr6	31558701	rs3130063	5	
chr6	31386130	rs3132472	5	
chr6	31428919	rs3130477	5	
chr6	31431812	rs3130907	5	POLR2A
chr6	31434330	rs3131619	5	
chr6	31434365	rs3094013	5	POLR2A
chr6	31434519	rs3094012	5	POLR2A
chr6	31434620	rs3131618	5	POLR2A
chr6	31308475	rs2854019	5	TRIM28
chr6	31313366	rs7450305	5	
chr6	31315628	rs9265965	5	
chr6	31315648	rs9265967	5	
chr6	31315662	rs9265968	5	
chr6	31316233	rs9265985	5	FOXA1
chr6	31316612	rs9265998	5	
chr6	31316694	rs9266001	5	
chr6	31321266	rs2596500	5	POLR2A
chr6	31044462	rs3094671	5	
chr6	31094702	rs3130557	5	
chr6	32342118	rs73729220	5	FOSL2, FOXA1
